# Cubic Mesocrystal Magnetic Iron Oxide Nanoparticle Formation by Oriented Aggregation of Cubes in Organic Media: A Rational Design to Enhance the Magnetic Hyperthermia Efficiency

**DOI:** 10.1021/acsami.3c03254

**Published:** 2023-06-30

**Authors:** David Egea-Benavente, Carlos Díaz-Ufano, Álvaro Gallo-Cordova, Francisco Javier Palomares, Jhon Lehman Cuya Huaman, Domingo F. Barber, María del Puerto Morales, Jeyadevan Balachandran

**Affiliations:** † Department of Immunology, and Oncology and Nanobiomedicine Initiative, Centro Nacional de Biotecnología (CNB-CSIC), Darwin 3, 28049 Madrid, Spain; ‡ Department of Nanoscience and Nanotechnology, Instituto de Ciencia de Materiales de Madrid (ICMM-CSIC), Sor Juana Inés de La Cruz 3, 28049 Madrid, Spain; § Graduate School of Environmental Studies, Tohoku University, 6-6-20 Aramaki aza aoba, Aoba-ku, Sendai 980-8579, Japan

**Keywords:** mesocrystals, thermal decomposition synthesis, cubic shape control, magnetic hyperthermia therapy, drug carrier

## Abstract

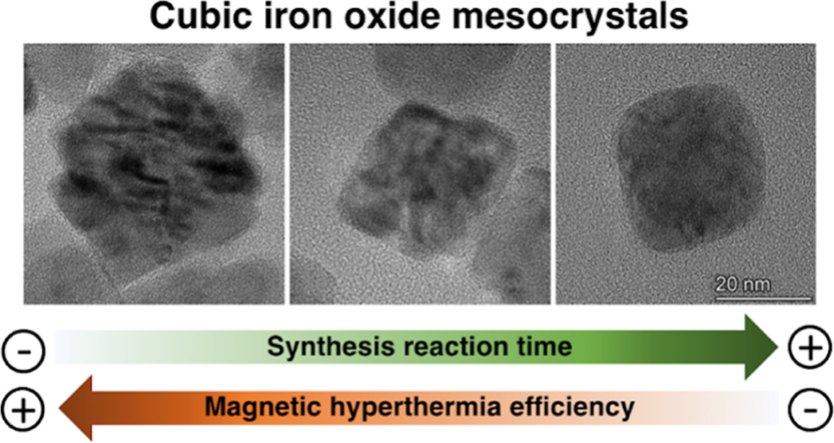

Magnetic iron oxide mesocrystals have been reported to exhibit collective magnetic properties and consequently enhanced heating capabilities under alternating magnetic fields. However, there is no universal mechanism to fully explain the formation pathway that determines the particle diameter, crystal size, and shape of these mesocrystals and their evolution along with the reaction. In this work, we have analyzed the formation of cubic magnetic iron oxide mesocrystals by thermal decomposition in organic media. We have observed that a nonclassical pathway leads to mesocrystals via the attachment of crystallographically aligned primary cubic particles and grows through sintering with time to achieve a sizable single crystal. In this case, the solvent 1-octadecene and the surfactant agent biphenyl-4-carboxylic acid seem to be the key parameters to form cubic mesocrystals as intermediates of the reaction in the presence of oleic acid. Interestingly, the magnetic properties and hyperthermia efficiency of the aqueous suspensions strongly depend on the degree of aggregation of the cores forming the final particle. The highest saturation magnetization and specific absorption rate values were found for the less aggregated mesocrystals. Thus, these cubic magnetic iron oxide mesocrystals stand out as an excellent alternative for biomedical applications with their enhanced magnetic properties.

## 1 Introduction

Mesocrystals are defined as a kind of multicore particles formed by an ordered assembly of smaller crystals of similar size and shape, unlike polycrystals that result in a random aggregation of crystals.^1,2^ They have gained increasing interest due to their emerging functionalities for various applications: photocatalysis, lithium-ion batteries, electrodes, gas sensors, optoelectronics, and nanomedicine.^3,4^ Several desirable properties can be obtained with a mesocrystal architecture; however, they cannot be reached for the same material in amorphous or single crystalline forms or as an unordered polycrystalline aggregate. For example, magnetic iron oxide nanoparticles (MNPs) with superparamagnetic properties are interesting alternatives for biomedical applications due to their biocompatibility and stability under physiological conditions.^5,6^ However, these NPs of around 10 nm have low magnetization to be separated or controlled by magnetic fields. Increasing the size of the nanocrystals can increase their saturation magnetization, but the dispersion of nanocrystals in an aqueous medium becomes an issue since the superparamagnetic–ferromagnetic transition occurs at a domain size of ∼30 nm for magnetite.^7^ On the contrary, magnetic mesocrystals with a high specific surface area^8^ provide high saturation magnetization and high water dispersibility which makes them ideal candidates for practically all biomedical applications. For example, in addition to the elevated surface area for drug delivery, their collective magnetic behavior leads to enhanced heating capabilities under alternating magnetic fields (AMFs) that exceed up to 1 order of magnitude that of single-core particles under the same magnetic field and frequency conditions.^9^ Despite the interest in this material, there is no universal mechanism to fully explain the formation pathway that determines the particle diameter, crystal size, and shape of the mesocrystals and their evolution along with the reaction to tune the structural and magnetic features by an appropriate choice of synthesis conditions.

Several synthesis routes have been described for the preparation of multicore iron oxide nanostructures, but many of them are based on the random clustering of individual NPs.^10,11^ On the contrary, mesocrystals are composed of spherical-shaped aggregates of crystallographically oriented primary particles. They are formed by nonclassical crystallization mediated by small NPs rather than single atoms, ions, or molecules like in the classical pathway.^12^ In all cases, magnetite mesocrystals are formed through a multistep process in aqueous media^13^ or nonaqueous media (polyol or organic media) in the presence of different molecules such as polyacrylic acid,^14,15^ sodium acetate and poly(vinylpyrrolidone),^16^*N*-methyl diethanolamine,^17^ poly(phenylenepyridyl) dendron and dendrimer^18^ citric acid,^19^ trioctylphosphine oxide (TOPO),^20^ or calixarenes,^21^ among others.

In polyol media, for example in diethylene glycol (DEG), using *N*-methyl diethanolamine as a cosolvent, green rust is formed first, which then transforms into the initial nuclei that agglomerate to form the primary crystals (10–20 nm). Finally, the aggregation of these magnetite crystals produces the final mesocrystalline structure, only if desorption and/or the decomposition of the DEG take place.^17^ Recently, it was shown in CoO mesocrystals that the adsorption of DEG molecules mainly occurs at the top of the {100} surface Co atoms. This DEG adsorption favors the formation of intermolecular hydrogen bonds between different CoO nanocrystals covered with DEG, developing oriented clusters. Then, a collective DEG departure occurs from the crystal surface, and a pseudo-single crystal is obtained due to interactions between different crystals that are thermodynamically more favorable than the interactions between DEG and CoO crystal surfaces.^22^

In organic media, only a few studies have shown the formation of iron oxide mesocrystal structures, using molecules such as TOPO^20^ or calixarenes that stabilize intermediate reaction stages, which facilitate the formation of these mesocrystal structures before they are finally transformed into large single NPs.^21^ However, in the presence of other dispersing agents, mainly surfactants such as oleic acid (OA), the aggregation process is hampered, and single crystals grow by diffusion.^23^ Single crystals’ shape can be controlled by the adsorption of certain molecules on specific crystal faces,^24^ leading to cubes, for example, when using carboxylic acids.^25^ However, in the case of mesocrystals, shapes other than spherical (e.g., cubic) are yet to be achieved, at least directly in one-step synthesis.^26−28^

It should be noted that multicore systems and nanocubes have been described as improved nanomaterials for magnetic hyperthermia, one of the most promising therapeutic approaches against cancer diseases, due to their specific absorption rate (SAR) values. Magnetic hyperthermia therapy is based on the accumulation of magnetic NPs in the tumoral-target site, followed by the application of AMFs. Magnetic NPs, specifically superparamagnetic iron oxide NPs, have the intrinsic ability to respond to AMFs by heat release, and the development of rationally improved MNPs plays a crucial role in the consolidation of this therapy and will allow it to reach high temperatures at lower doses.^29^ Specifically, improving the anisotropy of the MNPs and consequently enhancing their heat capacity can be achieved by shape control of the NPs, as is the case for cubic shapes^30,31^ or forming multicore NPs.^32^

In this work, we analyzed the formation of cubic iron oxide crystals and mesocrystals by thermal decomposition in organic media. The pathway that leads to the formation of mesocrystals was studied as a function of the solvent and the reaction time. Physicochemical characterization of the particles was carried out before and after transferring them to aqueous media by ligand exchange. Finally, the magnetic properties and heating capabilities of the aqueous suspensions dispersing single-core to multicore structures were measured at different frequencies and magnetic field intensities. Then, the doxorubicin loading by the electrostatic union between positive charges of doxorubicin amino groups and NP negative coating was evaluated using the most promising material. Although other strategies for doxorubicin loading in magnetic nanocarriers have been tested,^33,34^ electrostatic adsorption has been demonstrated to be stable at physiological pH, despite an initial burst release of around 15%^35,36^ or even more in the case of smaller NPs.^37^ It will be shown that achieving a rational MNP design allows the optimization of nanomaterials for each approach and consequently for their potential application.^38^

## 2 Experimental Section

### 2.1 Materials

Iron(III) acetylacetonate (Fe(acac)_3_; 97.0%), OA (90.0%, technical grade), biphenyl-4-carboxylic acid (95.0%), diphenyl ether (DPE; 98.0%, for synthesis), dibenzyl ether (DBE; 98.0%, purum), 1-octadecene (OCT; 90.0%, technical grade), toluene (99.5%, ACS reagent), *meso*-2,3-dimercaptosuccinic acid (DMSA; 98.0%), and dimethyl sulfoxide (DMSO; 99%.0) were obtained from Sigma-Aldrich.

### 2.2 MNPs Synthesis

MNPs were synthesized by the thermal decomposition method based on the procedure described by Mamiya et al.^39^ but with several modifications. In brief, 1060 mg of iron(III) acetylacetonate (3.0 mmol), 396.44 mg of biphenyl-4-carboxylic acid (2.0 mmol), and 2260 mg of OA (8.0 mmol) were dispersed in 20 mL of DPE (126.1 mmol), DBE (105.2 mmol), or OCT (62.5 mmol) in 100 mL round-bottom glassware. The mixture was heated up to the boiling point of the solvent at a rate of 10 °C/min under constant N_2_ flow (9 L/h) and mechanical stirring (500 rpm). After 30 min, the heating mantle was switched off and the suspension was left to cool down to room temperature. The MNPs were recovered by centrifugation (8000 rpm, 10 min; Sigma Centrifuge 4–15, rotor 12165-H) and washed with ethanol until complete removal of the organic waste. Finally, the MNPs were redispersed in toluene. Samples obtained in OCT were collected at different reaction times upon reaching the boiling temperature (30, 60, and 120 min) and named MC-30, MC-60, and MC-120, respectively.

### 2.3 MNPs Surface Coating

MNPs were coated with DMSA according to the procedure reported by Luengo et al.^40^ A solution containing 60 mg of DMSA (0.329 mmol) in 5 mL of DMSO was mixed with 20 mL of toluene containing 25 mg of MNPs (MNPs’ final concentration = 1.0 mg/mL). Then, the above mixture was agitated in a laboratory tube rotator for 3 days until surface-modified MNPs were no longer soluble in the organic mixture. Then, the supernatant was discarded, and the resulting precipitate was washed with ethanol by centrifugation (8000 rpm, 15 min) three times and then redispersed in distilled water. The dispersion was adjusted with KOH to pH 10 and dialyzed through a tubing cellulose membrane (typical molecular weight cutoff = 14,000 Da) for 3 days in distilled water with periodic water changes to remove unreacted DMSA and other impurities. Finally, the pH of the dispersions was adjusted to 7, concentrated using Amicon filter tubes (MWCO 10 kDa), and filtered using a 0.22 μm pore size membrane.

### 2.4 MNPs Characterization

The particle size, shape, and distribution were determined by transmission electron microscopy (TEM). Images were captured using a 100 keV JEOL-JEM 1010 microscope equipped with a Gatan Orius 200 SC digital camera working at an acceleration voltage of 60 kV. The mean particle size and size distribution were evaluated by measuring the largest internal dimension of at least 200 particles with the software ImageJ (NIH, USA), followed by data fitting to a log-normal distribution. MNPs’ plane orientations and the degree of fusion between internal cores were analyzed by using a scanning transmission electron microscope equipped with a high-angle annular dark-field detector (Thermo Fisher Talos F200X, 200 kV S/TEM). The crystal structure of the particles was identified by X-ray diffraction (XRD) performed using a Bruker D8 ADVANCE diffractometer with Cu Kα radiation, scanning between 2θ values ranging from 10 and 70°.

X-ray photoelectron spectroscopy (XPS) was used to characterize the surface chemistry of the samples and the oxidation state of Fe. The experiments were performed in a UHV chamber with a base pressure of 10–10 mbar, equipped with a hemispherical electron energy analyzer (SPECS Phoibos 150 spectrometer) and a 2D delay-line detector (Surface Concept). A nonmonochromatic X-ray source of Al Kα radiation (1486.6 eV) operated at 300 W was used. XPS spectra were recorded at the normal emission take-off angle, using 0.50 and 0.10 eV energy steps and 40 and 20 eV pass energies for survey spectra and detailed core level regions (Fe 2p, O 1s, C 1s, and Fe 3p), respectively. The binding energy is referenced to the C 1s photoelectron peak at 284.5 eV, CasaXPS software (Casa Software Ltd., Cheshire, UK) was used for data processing, and spectra were corrected by subtraction of the contribution of the Al Kα satellite emission.

The iron concentration of the magnetic colloids was measured by elemental analysis with inductively coupled plasma optical emission spectroscopy (ICP–OES, Plasma Emission Spectrometer ICP PerkinElmer mod. OPTIMA 2100 DV, PerkinElmer, Waltham, MA, USA). The samples (20 μL) were digested in 1 mL of aqua regia at 90 °C overnight and diluted up to a volume of 20 mL with Milli-Q water. Colloidal characterization was performed by dynamic light scattering (DLS) using a ZetaSizer Nano ZS (Malvern). Hydrodynamic (HD) size values (in intensity and number) and ζ-potential (ζ-pot) as a function of pH were the result of three different measurements at 25 °C. Infrared spectra were recorded in a spectrophotometer Bruker Vertex 70V in KBr pellets (2% wt of the sample).

Magnetic behavior as a function of the magnetic field (±7 T) and temperature was analyzed using a SQUID magnetometer (Quantum Design MPMS-3). MNP aqueous suspensions (50 μL) of known concentration were dried in a cotton piece and pressed in a sample holder. Hysteresis parameters such as saturation magnetization (*M*_s_) and coercivity (*H*_c_ = (*H*_c left_ – *H*_c right_)/2) were obtained at room temperature and 5 K, before (zero field cooling, ZFC) and after (field cooling, FC) applying a magnetic field of 5 T. The presence of exchange bias due to the existence of an antiferromagnetic phase was evaluated from the shift of the hysteresis loops after FC, so *H*_exch_ = *H*_left_ – *H*_c_.

### 2.5 MNPs Heating Efficiency and Doxorubicin Loading and Release

The heating efficiency of the MNPs in toluene and water under AMFs was analyzed in Eppendorf tubes containing 0.5 mL of the sample at a 1 mg/mL Fe concentration. The equipment was a Five Celes MP 6 kW device consisting of a generator with a frequency range between 100 and 400 kHz, connected to a cooling water circuit, a magnetic coil (magnetic field range: 0–60 mT) molded solenoid with an internal diameter of 71 mm, a capacitor box Type ALU CU, and an optic fiber sensor (measurement range from −40 to 200 °C). The Eppendorf tube was located inside a polystyrene cylinder cavity, which guaranteed a fixed position at the center of the magnetic coil and thermal isolation. The temperature change was measured as a function of time (d*T*/d*t*), and the linear slope between the 10 and 40 initial seconds was used to evaluate the heating efficiency in terms of SAR, power dissipation per unit mass of an element (W/g), using the following formula
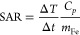
where *C*_*p*_ is the specific heat capacity of the liquid solvent (*C*_*p* toluene_ = 1590 J/kg·K and *C*_*p* water_ = 4185 J/kg·K) and *m*_Fe_ is the iron content per unit mass of the material solutions.

Doxorubicin loading and release were performed for the sample MC-30. Different pH load conditions (pH = 3.0, 5.0, 7.0, and 9.0), doxorubicin amounts (25, 50, 100, and 200 mg per mg of MC-30), and incubation times (15, 30, 60, 120, and 960 min) were tested. For the doxorubicin load, 1 mL of MC-30 at 100 μg/mL was incubated with doxorubicin in an orbital shaker. MNPs were centrifuged at 10,000 rpm for 10 min, and the doxorubicin concentration in the supernatant was measured in the fluorometer VICTOR2 (PerkinElmer) (λ excitation = 470 nm and λ emission = 585 nm). On the other hand, doxorubicin release was tested at pH = 3.5, 5.0, and 7.0 and the following experiment was carried out: after incubation, MC-30 was centrifuged (10,000 rpm for 10 min), the first supernatant was discarded (containing unbound doxorubicin), the pellet was resuspended and again centrifuged (several times), and the subsequent supernatant was analyzed using a fluorometer.

## 3 Results and Discussion

It is challenging to detect mesocrystals because the crystallographic fusion of their subunits may be a rapid process leading to the transformation of a mesocrystal to a single crystal. Here, for the synthesis of cubic iron oxide mesocrystals by thermal decomposition in organic media, three different solvents with increasing boiling temperatures from DPE (258 °C) to DBE (298 °C) and OCT (315 °C), were tested using iron(III) acetylacetonate as the precursor and biphenyl-4-carboxylic acid as the capping ligand. The rest of the synthesis conditions such as heat ramp, reaction times, stirring speed, and N_2_ flow remained unchanged.

The surfactant has been selected to guide the synthesis toward a specific shape or morphology.^23^ Specifically, cubic-shaped magnetite NPs have been already prepared from iron(III) acetylacetonate in high-boiling-temperature solvents, such as benzyl ether or DBE, in the presence of biphenyl-4-carboxylic acid due to its selective adhesion on the {100} facets.^25,39^ Trioctylphosphine^41^ and chloride ions^42^ are other ligands that selectively bind to {100} facets, also inducing a cubic shape. Complementarily, the selection of solvents with different boiling points allows the synthesis of NPs with different sizes.^43,44^ Carrying out the reaction at higher temperatures affects both the nucleation and the growth of the NPs by prompting the formation of a smaller number of nuclei in a shorter time that will grow further and also facilitating the diffusion of species from the solution to the surface of the NPs, obtaining larger NPs.^23^ On the other hand, the decomposition of the solvents in products, such as benzaldehyde (from DBE), may act as a shape-directing agent, leading to cubic-shaped NPs^45^ unchanged.

The results are presented in two sections, one focused on the formation and characterization of the single NP and mesocrystals and the effect of the different experimental parameters and the second part focused on the transference of the particles to water and the characterization of the colloids and their evaluation as heating and drug delivery agents.

### 3.1 Cubic Mesocrystal Formation

#### 3.1.1 Effect of the Solvent and the Surfactant Agent

**Figure 1:**
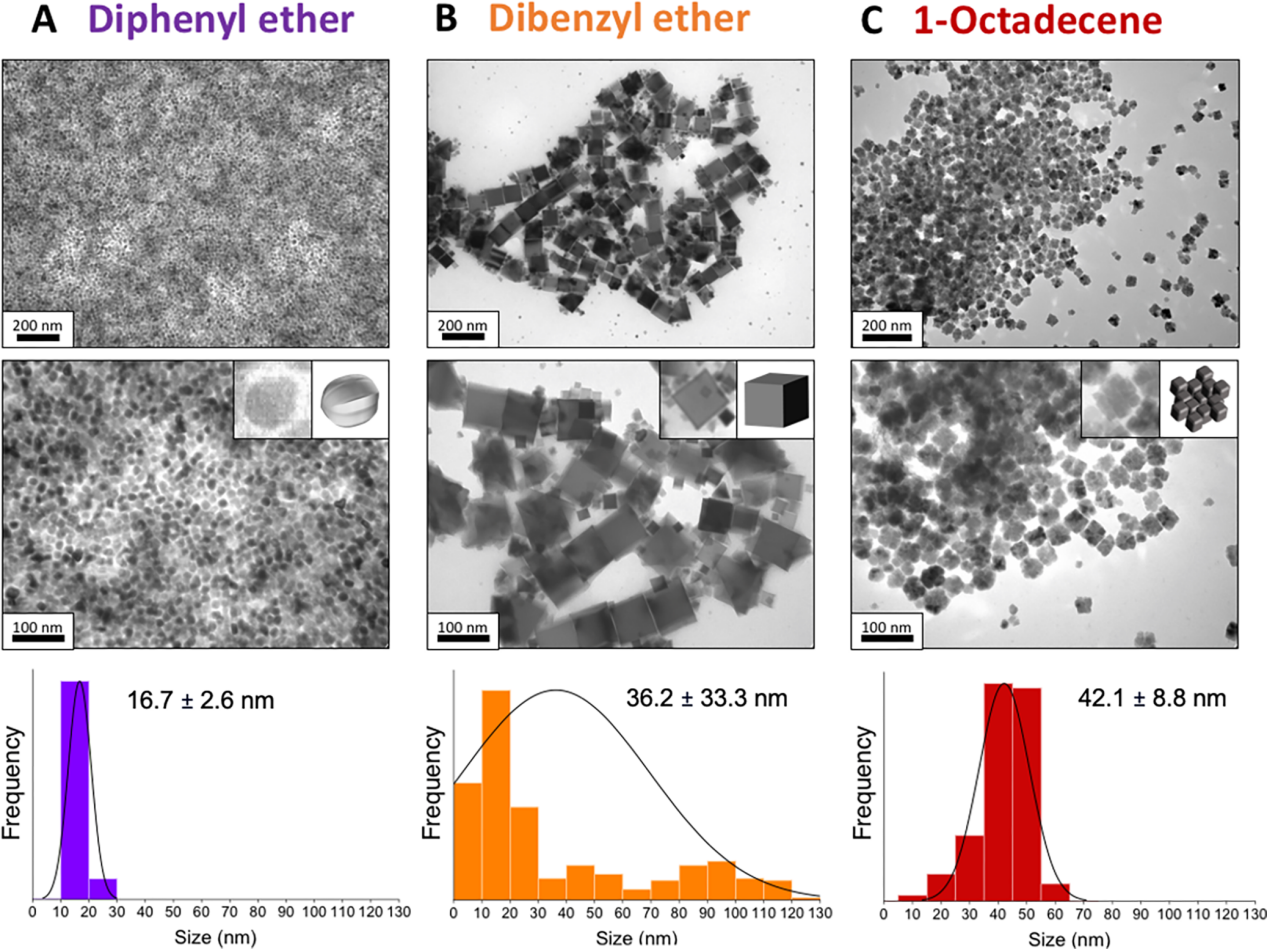
TEM images of MNPs obtained with different solvents at two different magnifications: (A) DPE, (B) DBE, and (C) OCT; from top to bottom: scale bar = 200 and 100 nm. Insets show the TEM image of a NP and its schematic representation. At the bottom, size distribution histograms are included.

TEM image analysis shows uniform particles with a narrow size distribution in DPE and OCT, while a wide size distribution was found for the particles prepared in DBE (Figure 1). The particle size increased from 16.7 ± 2.6 to 36.2 ± 33.3 and 42.1 ± 8.8 nm as the solvent boiling point increased from 258 °C (DPE) to 298 °C (DBE) and 315 °C (OCT). XRD analysis suggested that magnetite NPs have been obtained in all cases regardless of the solvent used (Figure S1A). All peaks correspond to an inverse spinel structure, without secondary phases such as metal iron or wüstite. The NP shape goes from nearly spherical to cubes when the solvent is changed from DPE to DBE, according to the formation of benzaldehyde products by the decomposition of DBE, as mentioned before.^45^ Surprisingly, the OCT solvent seems to be critical for obtaining cubic mesocrystals in contrast with DPE and DBE, where single-core NPs were produced (Figure 1). Some researchers have already shown the formation of mesocrystal structures in OCT, although in those cases, the final morphology was spherical.^21,46^

Here, we observed that OCT together with biphenyl-4-carboxylic acid act in a synergistic way, leading to the formation of cubic mesocrystals. It seems that the use of OCT, with a high boiling temperature (315 °C), facilitates fast and short nucleation, producing a large number of cores of about the same size. These cores aggregate in an ordered manner most probably due to their magnetic moment, which infers anisotropy along the easy magnetization direction ({111} for magnetite). The role of biphenyl-4-carboxylic acid is to preserve the colloidal stability of the precursor against flocculation and interact with the initial nuclei of magnetite in an oriented way to form the cubes, as previously reported for DEG molecules,^22^ and finally detached from the MNPs’ surface, leading to an ordered aggregation of nanocrystals. This is a nonclassical crystallization mechanism of particle formation that can be followed by assembly of the oriented crystals and, finally, sintering to form large single crystals^47^ (Scheme S1). If the carboxylic acid is absorbed completely and irreversibly on the iron oxide surface, interactions that promote the aggregation of the primary crystals are banished and single crystals are formed, as is probably in the case of MNPs produced in diphenyl and DBE.^48^ In these cases, a classical mechanism of formation of single-core NPs seems to be more likely, where following nucleation, growth occurs by monomer diffusion through the solution.^49^ The differences in size between the samples synthesized in DPE and DBE evidence the effect of the solvent boiling temperature from 258 to 298 °C in the nucleation and growth process. In general, high initial concentration or supersaturation, low viscosity, and low critical energy barrier favor greater nucleation that will result in smaller NPs as those presented in Figure 1A in DPE.

#### 3.1.2 Cubic-Shaped Mesocrystal Formation: Effect of the Reaction Time

There are several studies to elucidate the formation mechanism of mesocrystals, but to date, there is no universal pathway to fully explain this. The principal problem with mesocrystal growth is that the results are very difficult to analyze due to the length of scales. In this sense, it is very unlikely that all possible formation mechanisms have been explored. Nonetheless, some growth scenarios have been identified and described in the literature: (1) alignment by organic matrixes, (2) alignment by physical forces, (c) crystalline bridges together with epitaxial growth and secondary nucleation, (3) alignment by spatial constraints, (4) alignment by oriented attachment, and (5) alignment by face-selective molecules.^12^

**Figure 2:**
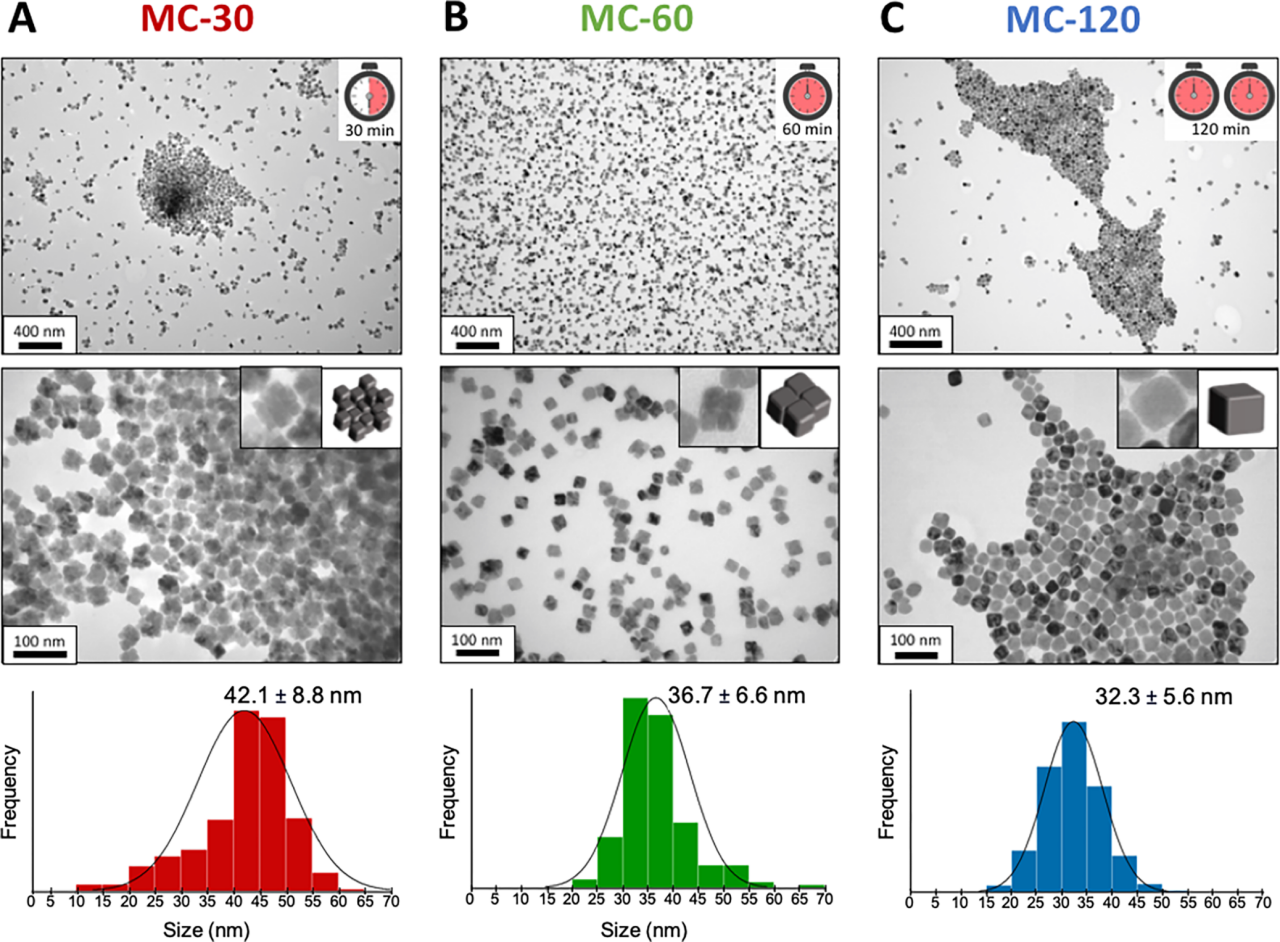
TEM images of the MNPs obtained at different reaction times: (A) 30 min (MC-30), (B) 60 min (MC-60), and (C) 120 min (MC-120). From top to bottom: scale bar = 400 and 100 nm. Insets show the TEM image of a NP and its schematic representation. At the bottom, size distribution histograms are included.

Here, the evolution of the mesocrystal sample along with reaction time was investigated for the sample synthesized in OCT (Figure 1C) to analyze its transformation to a single crystal. Synthesis conditions were kept constant (heat ramp, stirring, N_2_ flow, solvent, and the other reagents involved) except for the reaction time, which was set at 30, 60, and 120 min after reaching the boiling temperature of OCT (samples MC-30, MC-60, and MC-120, respectively). The evolution of NP formation was followed by TEM analysis, and their images are shown in Figure 2. MC-30 resulted in a very homogeneous cubic multicore shape, with a size of 42.1 ± 8.8 nm as mentioned before. Each multicore was composed of 8–16 small cubic cores as shown in the inset in Figure 2A. Sample MC-60 consisted also of a cubic multicore structure, but each NP was composed of fewer (between 3 and 6) and larger cubes with a final particle size of 36.7 ± 6.6 nm (Figure 2B). Finally, a monodisperse single cubic core was obtained for the MC-120 sample, with a size of 32.3 ± 5.6 nm (Figure 2C). Therefore, the control of the reaction time is essential to obtain mesocrystals (Figure 2A,B), and they are converted to a single crystal with extended reaction time (Figure 2C). This decrease in size over reaction time could be explained by coalescence of the oriented crystals and finally their sintering, removing the gaps between the cores (Scheme S1).

**Figure 3:**
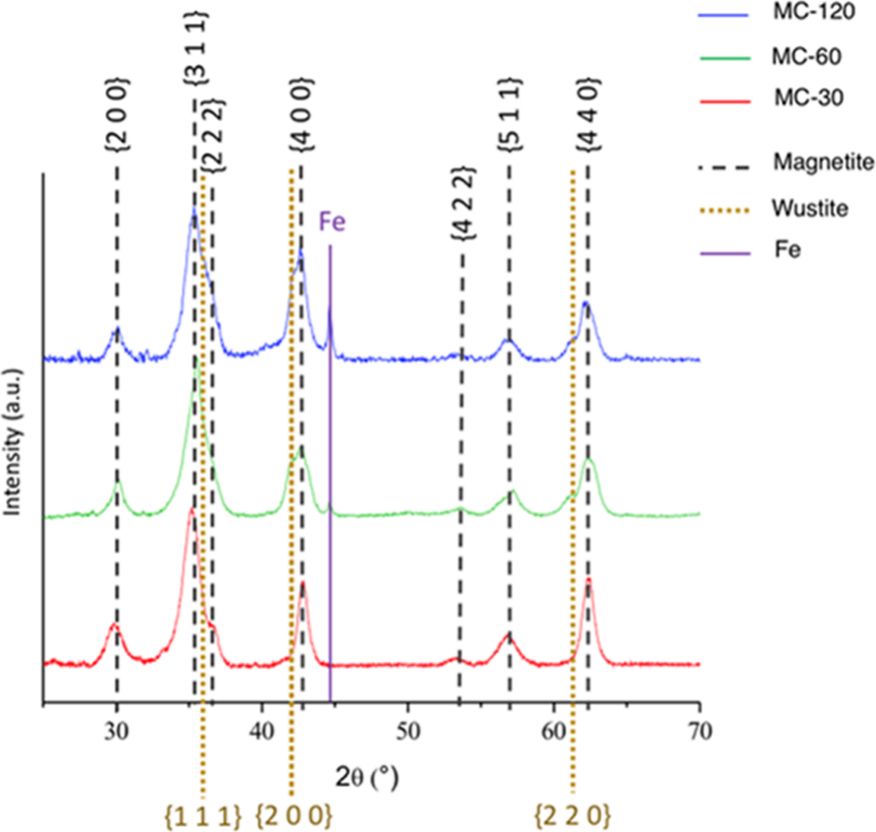
Powder X-ray diffractograms for MNPs synthesized in octadecene at different reaction times, 30, 60, and 120 min: MC-30 (red line), MC-60 (green line), and MC-120 (blue line). Dashed gray lines: identification of crystalline phases of magnetite. Yellow lines: identification of crystalline phases of wüstite. Purple line: Fe.

The crystal phases present in samples MC-30, MC-60, and MC-120 were analyzed using XRD (Figure 3). The main crystalline phase corresponds to magnetite in all three samples (dashed gray lines in Figure 3). A secondary phase present in samples MC-60 and MC-120 was identified as wüstite (yellow lines in Figure 3),^50^ together with a small fraction of metal iron inferred from the appearance of the diffraction line at 44° (2θ) (purple line in Figure 3), probably due to an over-reduction of the magnetite phase.^51^ The crystal size was extracted from the broadening of the magnetite {220} crystalline planes (to avoid interference with the wüstite phase). We observed that the crystal size for sample MC-30 was 7.6 nm, while for MC-60 and MC-120, it was 13.8 nm and 16.5, respectively.

It has been reported that the formation of magnetite by thermal decomposition in organic media is via the oxidation of wüstite, and it has been previously detected in relatively large magnetite NPs prepared under highly reductive environmental conditions, mainly with surfactants such as 1,2-hexadecanediol and solvents like OCT.^52^ Under these conditions, the oxidation rate is slower than the growth rate. Then, depending on the cooling rate and the particle size, the total conversion of wüstite and metal iron to magnetite may take place or a small fraction of these phases is preserved in the particle’s inner core. It should be mentioned that the amount of surfactants like 1,2-hexadecanediol is critical in such a way that higher amounts accelerate the decomposition of the iron precursor in the early stages of the reaction, the growth of the NPs is hampered by a low diffusion, and smaller NPs are obtained.^53^

**Scheme 1:**
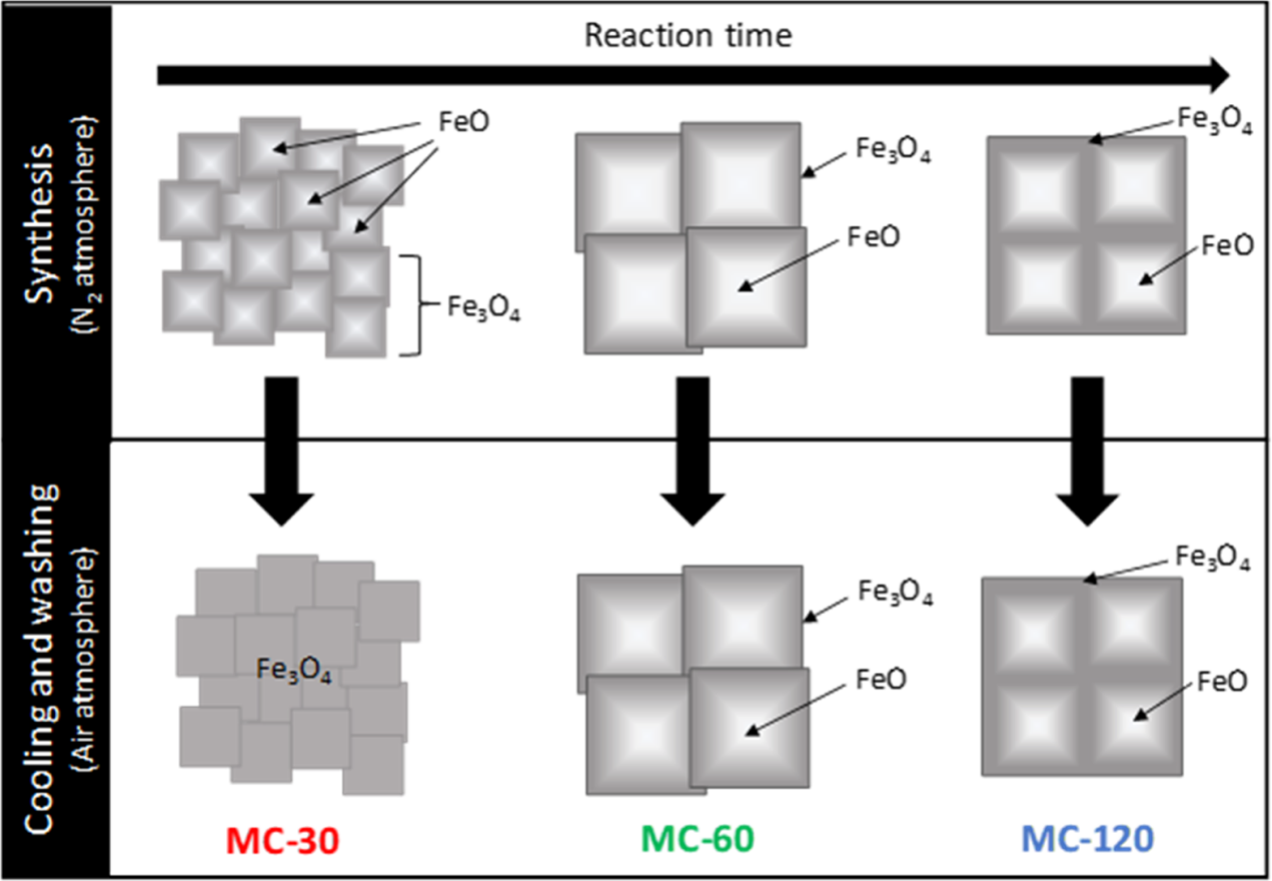
Mesocrystal Oxidation during Synthesis and Subsequent Cooling and Washing Processes Upper section: representation of the formation of wüstite inside the magnetite mesocrystals during the synthesis process (under a N_2_ atmosphere). Bottom section: representation of the oxidation process suffered by the NPs during cooling and washing (under an air atmosphere).

In our case, wüstite is detected for the longest reaction times probably due to the lack of oxidation inside the NPs when the cores are partially or totally fused, as observed for MC-60 and MC-120, respectively. In general, the oxidation reaction of the particles occurs from the surface, resulting in a core@shell structure having wüstite in the core and magnetite in the shell, as previously observed for large cubes synthesized from iron oleate^54^ and confirmed by elemental mapping.^55^ The process is illustrated in Scheme S2 for single-core particles. For mesocrystals, when the reaction is stopped after 30 min of reaction (MC-30), the intermediate wüstite is completely oxidized to magnetite during the cooling and washing process. However, the wüstite phase (and also the metal iron) was protected against oxidation in the largest nuclei (MC-60 and MC-120), as illustrated in Scheme 1. In fact, similar results have been observed by Muzzi et al. where they associate the formation of a core@shell FeO@Fe_3_O_4_ structure to the release of metal ions during the washing steps.^51^

#### 3.1.3 Structural Mesocrystal Characterization

**Figure 4:**
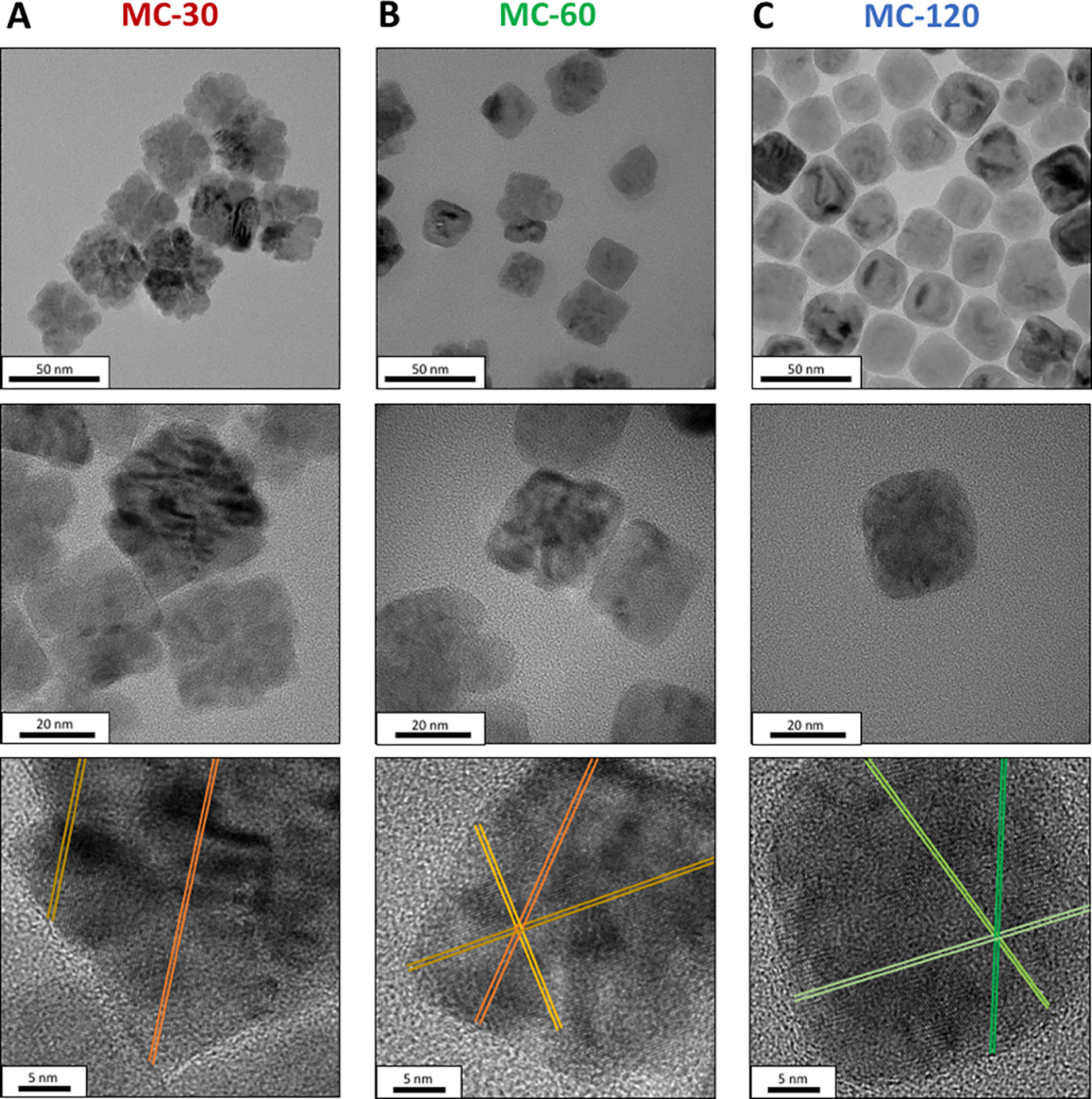
HRTEM images of MNPs synthesized in octadecene at different reaction times, 30, 60, and 120 min: (A) MC-30, (B) MC-60, and (C) MC-120 at different magnifications, increasing from the top to the bottom. Scale bar = 50, 20, and 5 nm. Images at the bottom show lines corresponding to crystallographic planes (yellow and orange lines indicate planes for magnetite and green lines for magnetite or wüstite).

Direct information on the crystalline structure of the NPs was obtained by high-resolution TEM (HRTEM) (Figure 4), showing the atomic lattice fringes along a particle that are used for the identification of the crystal phase (marked with different color lines in Figure 4, at the bottom). Mesocrystals are characterized by their high crystallinity due to the ordered attachment of the subunits, which makes it difficult to distinguish them from single crystals under electron diffraction (Figure S2). In fact, the planes from the individual cores that formed the multicore systems in MC-30 and MC-60 are continuous and, moreover, they cross these entire multicore NPs. Once the multicore has been fused in a large single core (MC-120), the continuity of the planes remains perfectly traceable.

The distance between the above-mentioned lattice fringes that corresponds to atomic spacings, that is, the interplanar distances, was measured for samples MC-30, MC-60, and MC-120 and compared with the interplanar distances of different iron oxides described by Cornell and Schwertmann^56^ (Table 1). In the case of MC-30, we observed interplanar distances of 0.2965 and 0.2424 nm, both corresponding to {220} and {222} planes of magnetite, respectively. For MC-60 and MC-120, the interplanar distances observed were 0.2899 and 0.2900 nm, respectively, which are close to the {220} plane of magnetite (0.2967 nm). Moreover, interplanar distances of 0.2517 and 0.2508 nm for MC-60 and MC-120, respectively, were related either to 0.2532 nm for the {311} plane of magnetite or 0.2490 nm for the {111} plane of wüstite (Table 1). These results are in good agreement with the XRD analyses (Figure 3), where the peak related to the magnetite plane {311} is widened for MC-60 and MC-120, probably due to its overlap with the peak corresponding to the {111} plane of wüstite.

**Table 1:** Summary of Interplanar Distances Corresponding to MC-30, MC-60, and MC-120 Assigned to the Type of Iron Oxide and the Crystal Plane According to Cornell and Schwertmann^56^

	interplanar distance observed (nm/plane)	iron oxide	crystal plane
MC-30	0.2965 ± 0.0085	magnetite	{220}
	0.2424 ± 0.0074	magnetite	{222}
MC-60	0.2899 ± 0.0044	magnetite	{220}
	0.2517 ± 0.0060	magnetite	{311}
		wüstite	{111}
MC-120	0.2900 ± 0.0082	magnetite	{220}
	0.2508 ± 0.0040	magnetite	{311}
		wüstite	{111}

### 3.2 Colloidal Suspensions and Biological Applications: Magnetic Hyperthermia Therapy

#### 3.2.1 DMSA Coating for Biological Purposes

**Scheme 2:**
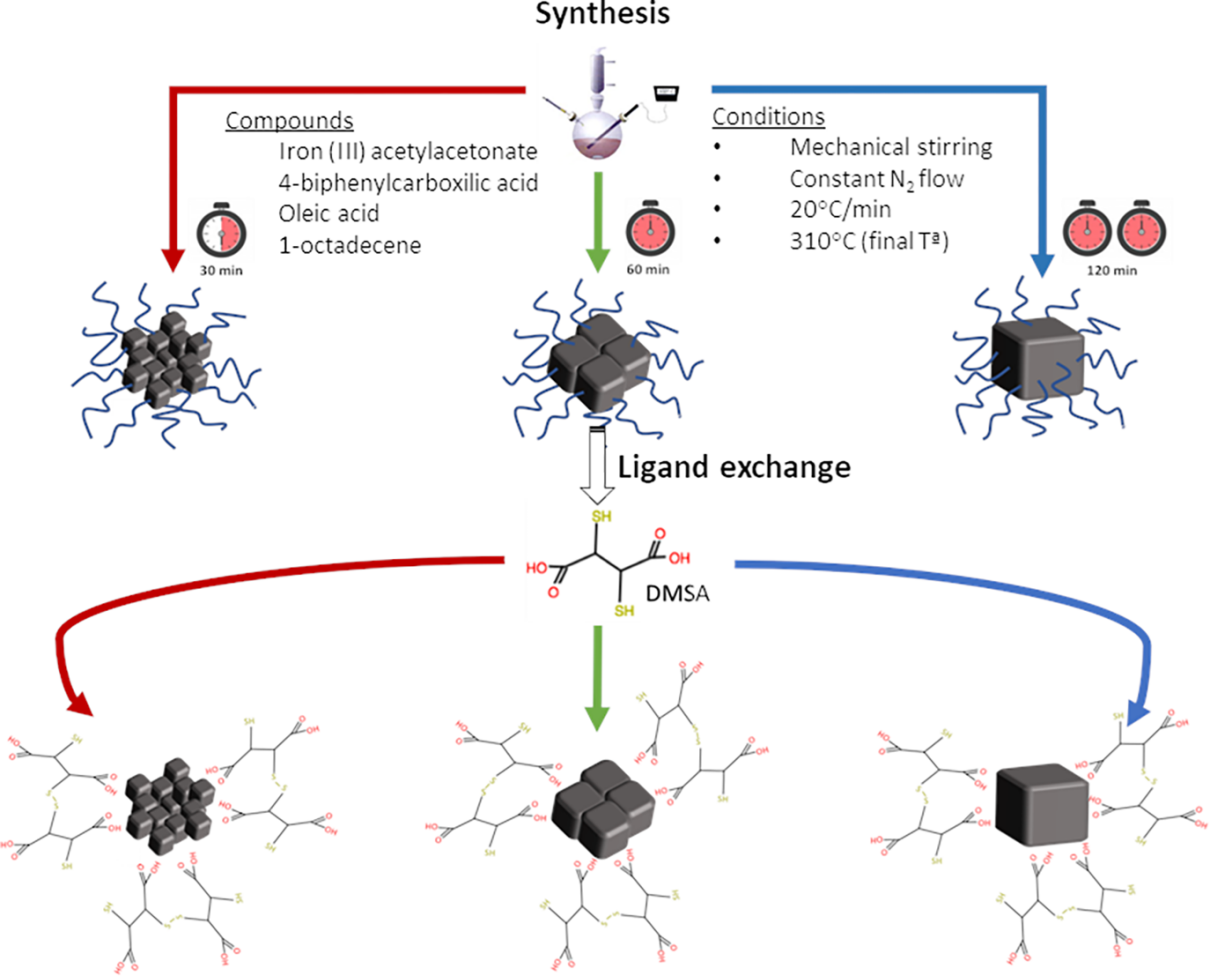
Representation of NP Synthesis and Transference to Water by Ligand Exchange with Dimercaptosuccinic Acid (DMSA)

NPs prepared in organic media are coated with OA but can be easily transferred to aqueous media by ligand exchange with DMSA, widely used for these purposes.^57−59^ Excellent colloidal stability in water was obtained in all cases without evident aggregation or sedimentation over a long period of time (days). HD sizes were measured by DLS in intensity and number and are included in Table 2 together with the ζ-potential. In all cases, the value of the HD sizes in intensity or number is around 109–134 or 45–51 nm, respectively, and the surface charge is around −20 mV at neutral pH, revealing the presence of DMSA carboxylic groups at the NP surface.^40^ A schematic workflow from NP synthesis to its transference to water by ligand exchange with DMSA is shown in Scheme 2, and TEM images for MC-30 in water are shown in Figure S3.

**Table 2:** MNP Characterization in Aqueous Media: HD Size and ζ-Pot

	HD size^a^		
	intensity (nm)	number (nm)	ζ-pot (mV)^b^
MC-30	134 ± 27	45 ± 9	–19.1 ± 1.5
MC-60	109 ± 25	49 ± 11	–20.4 ± 1.6
MC-120	118 ± 22	51 ± 10	–18.8 ± 1.4

a Error calculated through the polydispersity index; *n* = 3.

b Error calculated as standard deviation; *n* = 3.

**Figure 5:**
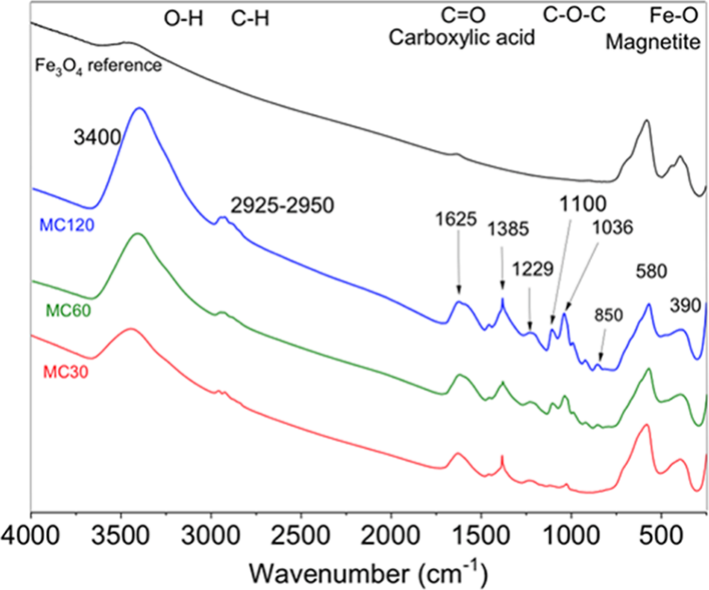
FTIR spectra for MC-30, MC-60, and MC-120 coated with DMSA. The main peaks were assigned as follows: 1625 and 1385 cm^–1^: COOH groups of DMSA. 1100 and 1036 cm^–1^: biphenyl carboxylic acid rests. 580 and 390 cm^–1^: magnetite (shoulders: maghemite and/or wüstite).

Infrared spectra showed bands at 580 and 390 cm^–1^ assigned to Fe–O vibration of magnetite, bands at 1625 and 1385 cm^–1^ corresponding to asymmetric and symmetric vibrations of DMSA carboxyl groups, respectively, bands at 1100 and 1036 cm^–1^ assigned to C–O–C vibrations of biphenyl carboxylic acid, and bands at 3400 and 2925–2950 cm^–1^ corresponding O–H of water and C–H molecules, respectively (Figure 5). Interestingly, the Fourier-transform infrared (FTIR) spectroscopy bands at 1100 and 1036 cm^–1^ assigned to biphenyl carboxylic acid increase in intensity as the reaction is extended probably due to the promotion of surfactant degradation. Partial oxidation of magnetite to maghemite as well as the presence of some wüstite cannot be discarded because of the existence of some shoulders in 580 and 390 cm^–1^ bands.^60^ In fact, XRD of the samples after transference to water reveals the presence of wüstite for MC-60 and MC-120 (Figure S1B). This process is represented in Scheme S3, and FTIR spectra for sample MC-30 before and after ligand exchange can be observed in Figure S4.

**Figure 6:**
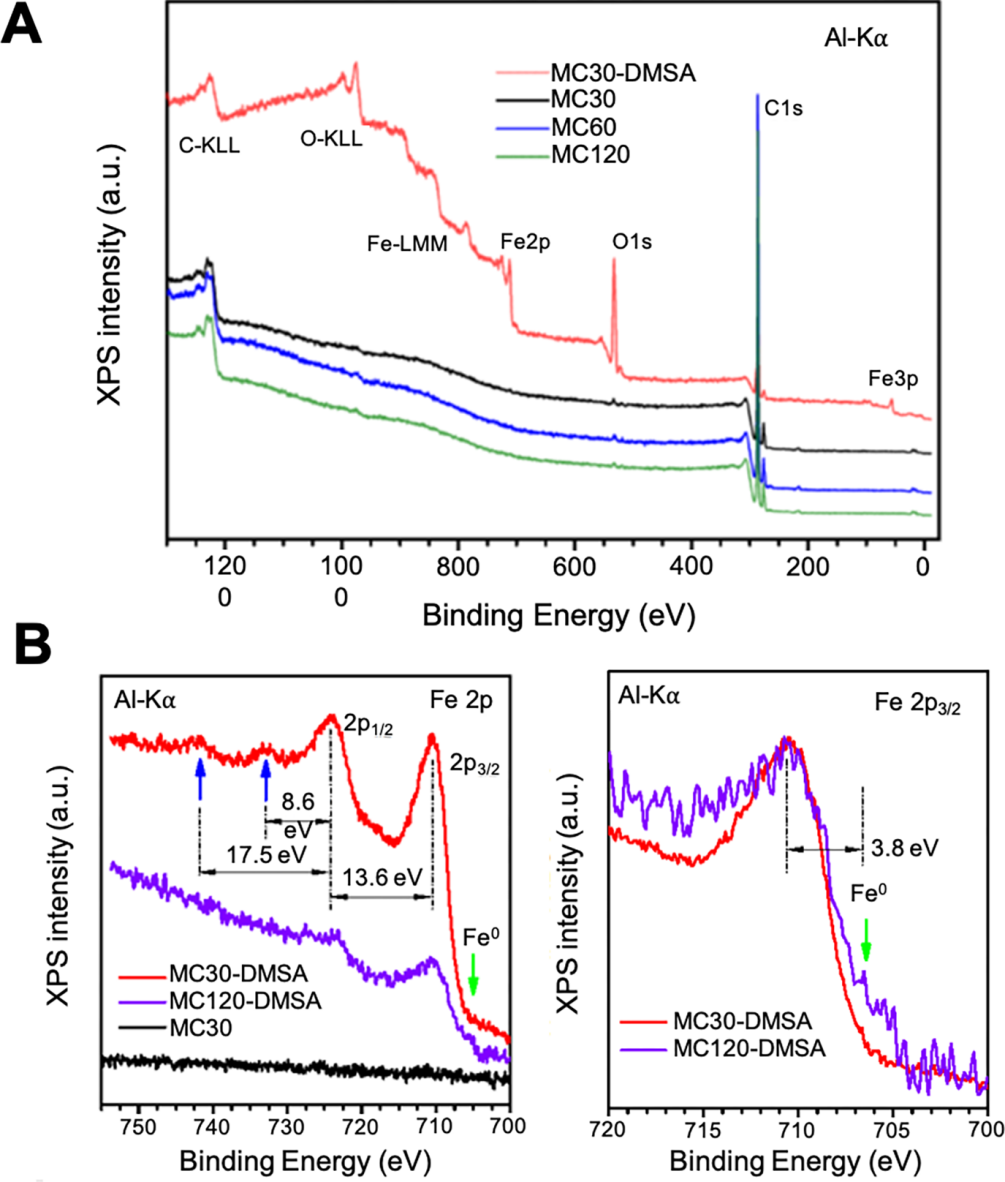
(A) XPS survey spectra corresponding to the mesocrystals in organic media (MC-30, 60, and 120) and in water (MC-30), where the main photoelectron lines and Auger transitions are labeled. (B) Energy region of the Fe 2p spectra corresponding to samples MC-30 in organic media and DMSA-coated MC-30 and MC-120. Blue arrows: energy splitting of the spin–orbit 3/2 and 1/2 doublet.

Additionally, XPS was used to characterize the surface chemistry of the samples before and after DMSA coating and the oxidation state of Fe. Figure 6A displays the XPS survey spectra corresponding to the mesocrystals in organic media (MC-30, 60, and 120) and in water (MC-30) where the main photoelectron lines and Auger transitions are labeled. Samples in organic media only exhibit an intense C 1s peak and its C-KLL Auger emission coming from the “organic shell” in which the MNPs are embedded and a weak O 1s emission due to the sample exposure to atmospheric pressure prior to XPS experiments. The thickness of this outer coating is higher than the probing depth of the technique limited to a few nanometers, which interferes with the analysis of the Fe-oxide phases present.

On the contrary, the DMSA-coated particle in water shows photoelectron peaks corresponding to the emission of Fe oxide. Figure 6B displays the energy region of the Fe 2p spectra corresponding to samples MC-30 in organic media and DMSA-coated MC-30 and MC-120. MC30 sample emission is equivalent for all the MC samples studied, and only the background signal coming from the inelastically scattered electrons is detected in the Fe 2p region. However, the DMSA-coated MC-30 sample spectrum shows the photoelectron emission of the complex line shape mainly dominated by two wide peaks corresponding to the spin–orbit 3/2 and 1/2 doublet. Their energy splitting and the presence/absence of characteristic satellites reveal two weak emissions (blue arrows in the figure) shifted to higher binding energies of 8.6 and 17.5 eV, respectively, above the 2p_1/2_ component and a negligible signal between the doublet. In accordance with this fact and together with the binding energy values of the component 2p_3/2_ (710.6 eV) and 2p_1/2_ splitting (13.6 eV), it is suggested that the surface oxide signal comes mainly from Fe_3_O_4_.^61,62^ Sample MC-120 coated with DMSA presents the characteristic peak on the right assigned to metal Fe (Figure 6B). A prominent emission at 706.7 eV, significantly shifted to lower binding energy than that of the main oxide peak, can be clearly attributed to 2p_3/2_ from metal iron, in accordance with the XRD result.

#### 3.2.2 Magnetic Properties

**Figure 7:**
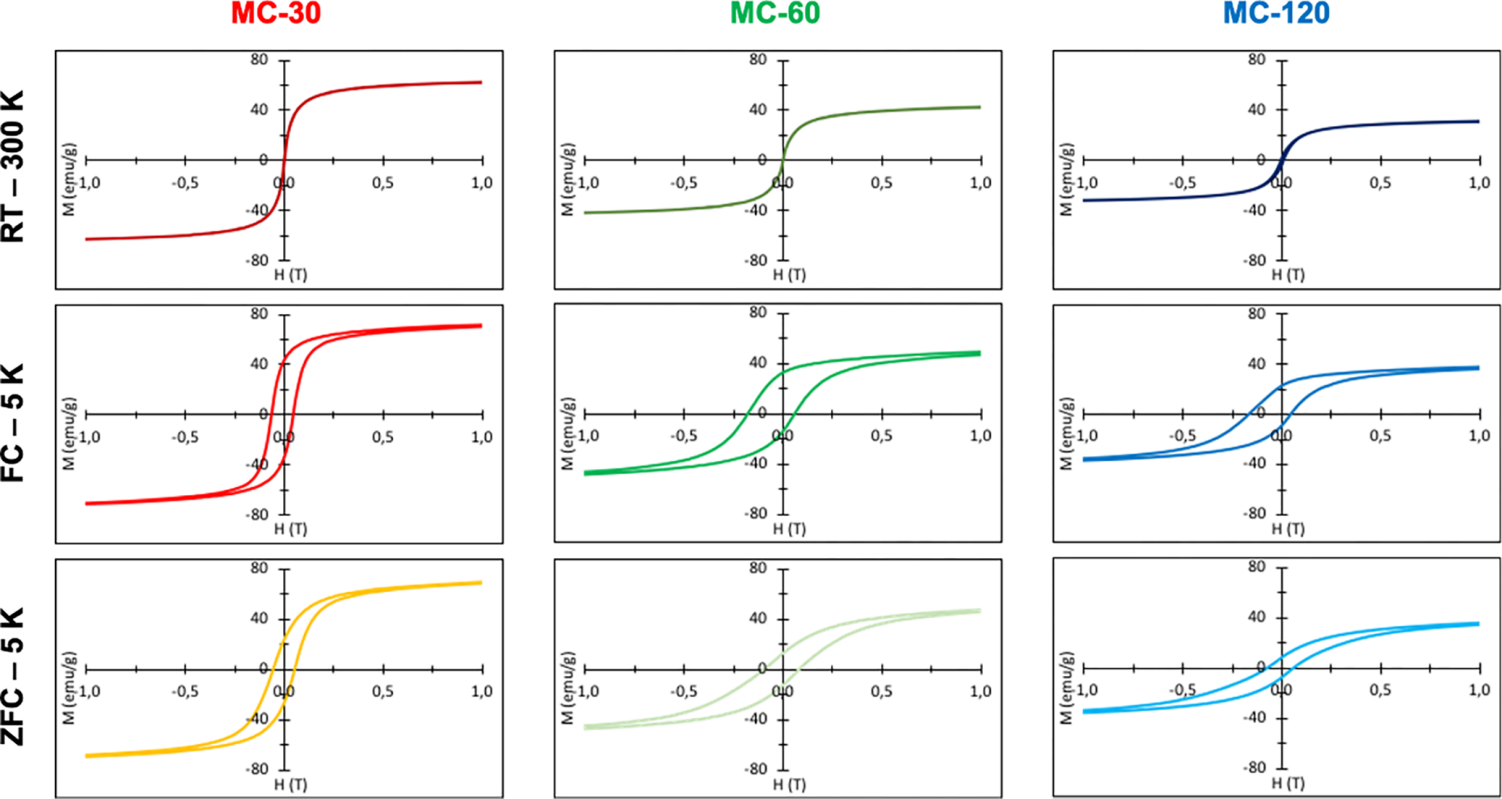
Hysteresis loops summarized for DMSA-coated MC-30, MC-60, and MC-120 under the conditions of RT–300 K, ZFC–5 K, and FC–5 K.

The magnetic properties of the aqueous suspensions were analyzed using a SQUID magnetometer. The hysteretic curves and magnetic parameters are shown in Figure 7 and Table 3. Magnetic measurements at room temperature showed the typical features expected for superparamagnetic NPs (zero coercivity and remanence) for samples MC-30 and MC-60, despite the large particle sizes (∼40 nm) and in good agreement with their mesocrystal structure. In contrast, sample MC-120 even with a slightly smaller particle size exhibited a ferromagnetic behavior at RT (*H*_c_ = 10.0 mT) because of its single-core character after coalescence. The saturation magnetization decreased from 63.3 emu/g (MC-30) to 46.1 emu/g (MC-60) and 31.6 emu/g (MC-120) (Figure S5A) probably due to the presence of the secondary antiferromagnetic phase wüstite in the last two samples, as shown by XRD (Figure S1B).

**Table 3:** Hysteretic Parameters for DMSA-Coated MC-30, MC-60, and MC-120 under the Following Conditions: RT–300 K, ZFC–5 K, and FC–5 K and 5 T^a^

	MC-30				MC-60				MC-120			
	*M*_s_ (emu/g)	*M*_r_ (emu/g)	*H*_c_ (mT)	*H*_exch_ (mT)	*M*_s_ (emu/g)	*M*_r_ (emu/g)	*H*_c_ (mT)	*H*_exch_ (mT)	*M*_s_ (emu/g)	*M*_r_ (emu/g)	*H*_c_ (mT)	*H*_exch_ (mT)
RT–300 K	63.3	0.0	0.0	0.0	46.1	0.0	0.0	0.0	31.6	2.7	10.0	0.0
ZFC–5 K	74.8	24.0	55.1	5.0	57.0	13.4	90.1	10.0	39.3	8.0	65.1	5.0
FC–5 K	75.7	43.8	55.1	10.0	59.1	35.6	120.2	60.1	40.0	22.5	112.6	57.6

a Saturation magnetization (*M*_s_), coercivity (*H*_c_), remanence (*M*_r_), and exchange coupling (*H*_exch_).

At low temperatures (ZFC), the decrease in saturation magnetization is preserved, indicating that the reduction in samples MC-60 and MC-120 is not due to a small fraction of NPs but due to the presence of the wüstite phase (Figures 7 and S5B). For MC-30, the saturation magnetization value at low temperatures was 75.7 emu/g, close to the bulk value of maghemite (ca. 76 emu/g at 273 K and ca. 83 emu/g at 5 K^63^), while the *M*_s_ values for MC-60 and MC-120 were 46.1 and 31.6 emu/g, respectively. The presence of wüstite as a minor secondary phase and some surfactant rest, as detected by FTIR spectroscopy (Figure 5), account for the reduction in *M*_s_ for samples MC-60 and MC-120. On the other hand, the coercivity increases from 55 mT for MC-30 up to 90 mT and 65 mT for MC-60 and MC-120, respectively. We cannot discard the presence of some rest of the metal Fe observed in the X-ray diffractogram for MC-60 and MC-120 and by XPS, although it is not reflected in the *M*_s_ values, indicating that the amount is very low.

Hysteresis loops at 5 K after field cooling (5 T) from 300 K exhibited a shift from the origin and significant coercivities (120 and 112 mT) for MC-60 and MC-120 (Table 3), indicating exchange interactions between two magnetic phases, one soft (the magnetite/maghemite, which provides the higher *M*_s_) and another hard (the wüstite, which brings the high *H*_c_) (Figures 7 and S5C). The exchange coupling (*H*_exch_) values are similar for samples MC-60 and MC-120 and practically unneglectable for MC-30, confirming the presence of a unique iron oxide phase in this mesocrystal made of the smallest cores. This behavior has been previously observed for core@shell and wüstite@magnetite NPs of large size (∼20 nm).^64^ In general, this phenomenon is usually observed in binary systems comprising antiferromagnetic (e.g., wüstite) and ferromagnetic (e.g., magnetite) ordered phases or mixtures of Fe_3_O_4_ and Fe_2_O_3_ that can be in core@shell or not core@shell structures, resulting in an increase of *H*_c_ and leading to a horizontal shift of the hysteresis loop, characterized by the exchange bias field.^51^

#### 3.2.3 Heating Efficiency under an AMF and Doxorubicin Adsorption and Release

The heating capability of MNPs can be diminished when they are transferred from organic media to water or a biological medium due to aggregation issues.^65^ Thus, the heating efficiency of the NP suspensions was evaluated in toluene and water under different magnetic fields and frequency conditions, and the respective SAR values were calculated.

**Figure 8:**
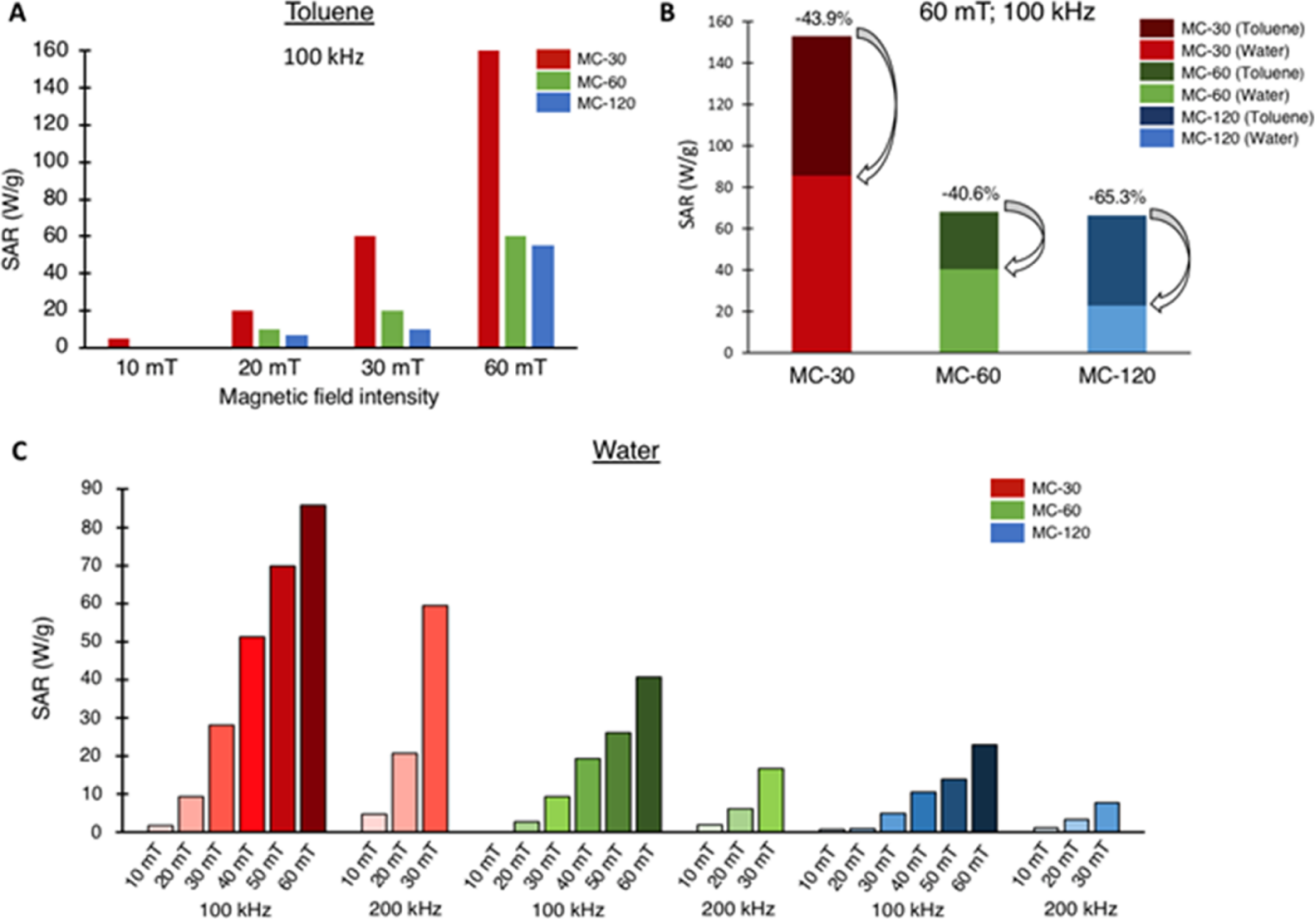
Heating efficiency (A) of MC-30 (red), MC-60 (green), and MC-120 (blue) in toluene. SAR values at 10–60 mT and a 100 kHz frequency. (B) SAR values of MC-30, MC-60, and MC-120 in toluene (dark colors) and water (light colors) at 100 kHz and 60 mT; numbers indicate the heating efficiency loss by medium transference (in %). (C) Heating efficiency of MC-30, MC-60, and MC-120 coating with DMSA in water. SAR values at 10–60 mT and 100 kHz and at 10–30 mT and 200 kHz.

In toluene, sample MC-30 exhibited significantly a higher SAR than the other MNPs in all field conditions tested (100 kHz and 10, 20, 30, and 60 mT, Figure 8A). These differences were marked at high magnetic field intensities (60 mT). Meanwhile, MC-60 and MC-120 showed a very similar response with SAR values slightly higher for MC-60.

After water transference, SAR values were analyzed under the field conditions where the MNPs in toluene presented the highest heating efficiency (60 mT and 100 kHz). In all cases, a decrease in SAR values was observed from around 150 W/g down to around 80 W/g for sample MC-30, which could be partly due to the higher specific heat capacity of water than that of toluene. The reduction in the SAR for MC-30 and MC-60 was around 40%, which corresponded to a reduction in terms of heating per time from 5.9 °C/min in toluene to 1.4 °C/min in water and from 2.6 °C/min in toluene to 0.6 °C/min in water, respectively. For sample MC-120, the decrease in SAR was around 65%, which means from 2.4 °C/min in toluene to 0.3 °C/min in water (Figure 8B).

Finally, a systematic SAR value screening at different magnetic field intensities (10, 20, 30, 40, 50, and 60 mT) and frequencies (100 and 200 kHz) was performed for the MNPs in water (Figure 8C) and the corresponding heating curves are shown in Figure S6. SAR values increased with the magnetic field frequency and intensity as expected, with no saturation for any of the MNPs. It is clearly seen how MC-30 presents the highest SAR values (90 W/g), followed far behind by MC-60 (40 W/g), and finally MC-120 (20 W/g) (Figure 8C). Probably, larger magnetic fields are required to achieve higher SAR values from sample MC-120 which presents a ferromagnetic behavior at room temperature as previously shown. However, the MC-30 mesocrystal is easily activated to produce heat at moderate magnetic fields, despite the large particle size and in agreement with its superparamagnetic behavior at room temperature. In addition, mild hyperthermia within the therapeutic windows (39–43 °C) could be reached using NP concentrations between 150 μg/mL and 500 μg/mL that produce temperature variations between 2.5 and 5.0 °C, respectively (Figure S6B).

Compared with other magnetic NPs tested in similar magnetic field conditions (frequency and intensity), we observed that SAR values for MC-30 are comparable to the values for single-core magnetite NPs of 14–18 nm prepared by thermal decomposition in organic media.^66^ Spherical mesocrystals of 56 nm have similar SAR values to those reported here but in higher frequency fields.^16^ Considering that the maximum SAR value is closely related to the optimal NP size,^67,68^ tuning the size of these cubic mesocrystals (MC-30) is expected to improve their heating performance. It should be emphasized that comparing SAR values is not an easy issue since there is no unique and universal protocol to calculate it.^69^ Different equipment, operating conditions, thermal isolations, concentrations,^70^ and so forth result in large SAR variations and difficulties to make a proper comparison between different laboratories.

Furthermore, taking advantage of the high specific surface area of mesocrystal-structured MC-30, the possibility of adsorbing a chemotherapeutic drug was tested using doxorubicin which is a well-established model^71^ that has demonstrated to produce a desirable synergic effect between hyperthermia and chemotherapy.^72,73^ The doxorubicin load in magnetic nanocarriers could follow different strategies,^33,34^ being the most common electrostatic union between positive charges of the doxorubicin amino groups and NP negative coating. Moreover, this electrostatic adsorption has been demonstrated to be quite stable at physiological pH^35,36^ and shows significant cytotoxicity in several cancer cell lines.^74^ Here, doxorubicin was bound to MC-30 NPs through the DMSA carboxyl group. We achieved an efficient (>75%) and large doxorubicin load in MC-30 (0.1 mg of doxorubicin/mg MC-30) without undesirable release at physiological pH after several washes for 72 h (<10%) (Figure S7). That is, using between 10 and 100 μg of particles, a concentration of doxorubicin of 1–10 μg will be reached, which corresponds to 1.8–18.4 μM that is within the IC_50_ for cancer cells.^75^ The loading efficiency of MC-30 mesocrystals was similar to other systems based on magnetic NPs^76^ but slightly lower than the loading into organic vesicles.^77^ Doxorubicin loading could be improved by functionalizing the MC-30 surface with MamC protein, for example, which showed a high loading (0.69 mg of doxorubicin/mg magnetite), low unspecific release at physiological pH (5%), and efficient desorption when the pH was changed to 5 and it was supported on magnetite NPs.^78^

## 4 Conclusions

Obtaining an orderly assembled mesocrystal is challenging and requires the optimization of several experimental parameters. In this work, we studied the formation of cubic mesocrystals through a rational synthesis design based on the capping agent, solvent, and reaction time as key parameters. First, crystal plane-specific adsorption of biphenyl-4-carboxylic acid leads to cubic shapes. Second, viscous and high-boiling-point OCT was used as a solvent to promote a cubic shape through a complete decomposition of reagents and core aggregation to minimize the energy of the system. Third, the reaction time was controlled to avoid the presence of wüstite that would hamper the magnetic properties and limit the range of applications.

The reaction mechanism governing this synthesis is explained by the nonclassical crystallization pathway, where crystal nucleation and a brief cubic-shape-like crystal growth occur at the initial stages. Then, minimization of the system energy drives core aggregation via an ordered assembly, yielding a cubic mesocrystal formed of several small cubic cores (8–16) at 30 min. As the reaction progresses, cubes are fused and the cubic mesocrystal is composed of 3–6 medium-sized cubic cores after 60 min. Finally, after 120 min, the mesocrystal is completely sintered and transformed into a large single cube. The cubic mesocrystal formation and the reaction mechanism proposed are mainly supported by (a) tracking by TEM after reaching the maximum reaction temperature at different times, (b) crystal size measurement through XRD, where MC-120 shows larger crystal size than the mesocrystals MC-30 and MC-60, and (c) the HR-TEM analysis, where the crystal planes between each one of the single cores which form MC-30 and MC-60 cross the entire multicore NP continuously, as observed in the single-core NP MC-120.

The rational design of NPs is a powerful approach to improve their applications in different areas such as magnetic hyperthermia therapy. Consequently, in this work, we demonstrate that besides controlling the shape of individual NPs or promoting the formation of multicore systems, we can combine both strategies to obtain mesocrystals with a guided shape. Moreover, the cubic mesocrystal coated with DMSA could combine their capacity as nanoheaters with other therapeutic approaches as a drug carrier, allowing a combined therapy. High adsorption and no undesirable release at pH 7 of the chemotherapeutic agent doxorubicin were successfully demonstrated.

## Supporting Information Available

Structural characterization of MNPs synthesized in DPE, DBE, and OCT and MC-60 and MC-120 after transfer to water by XRD; schematic representation of the crystallization pathways and the oxidation process of MNPs during synthesis and after the transfer to water; electron diffraction of MC-30, MC-60, and MC-120; TEM images of DMSA-coated MC-30 and MC-120; FTIR spectra of MC-30 before and after transfer to water; full hysteresis loops at RT–300 K, ZFC–5 K, and FC–5 K for DMSA-coated particles; temperature vs time heating curves under AMFs; doxorubicin loading and release conditions in detail; and calibration curve of doxorubicin (PDF)

## Abbreviations

MNPs: magnetic iron oxide nanoparticles
MC: mesocrystal
DPE: diphenyl ether
DBE: dibenzyl ether
OCT: 1-octadecene
DMSA: *meso*-2,3-dimercaptosuccinic acid
AMFs: alternating magnetic fields
SAR: specific absorption rate
