# Supplementary material for: Cubic Mesocrystal Magnetic Iron Oxide Nanoparticle Formation by Oriented Aggregation of Cubes in Organic Media: A Rational Design to Enhance the Magnetic Hyperthermia Efficiency
Source: ACS Appl Mater Interfaces. 2023 Jun 30;15(27):32162–76. doi: 10.1021/acsami.3c03254 (PMC10347427; doi:10.1021/acsami.3c03254)
Supplement: Supplementary file 1 — am3c03254_si_001.pdf [file am3c03254_si_001.pdf]

Supporting information

Cubic mesocrystal magnetic iron oxide nanoparticles formation by oriented aggregation of cubes in organic media: a rational design to enhance the magnetic hyperthermia efficiency

David Egea-Benavente[1], Carlos Díaz-Ufano[2], Álvaro Gallo-Cordova[2], Francisco Javier Palomares[2], Jhon Lehman Cuya Huaman[3], Domingo F. Barber[1], María del Puerto Morales[2], Jeyadevan Balachandran[3]**

[1]Department of Immunology, and Oncology and Nanobiomedicine Initiative, Centro Nacional de Biotecnología (CNB-CSIC), Darwin 3, 28049 Madrid, Spain

[2]Department of Nanoscience and Nanotechnology, Instituto de Ciencia de Materiales de Madrid (ICMM-CSIC), Sor Juana Inés de la Cruz 3, 28049 Madrid, Spain.

[3]Graduate School of Environmental Studies. Tohoku University, 6-6-20 Aramaki aza aoba, Aoba-ku, Sendai, 980-8579, Japan

*Correspondence: degea@cnb.csic.es and balachandran.jeyadevan.c2@tohoku.ac.jp

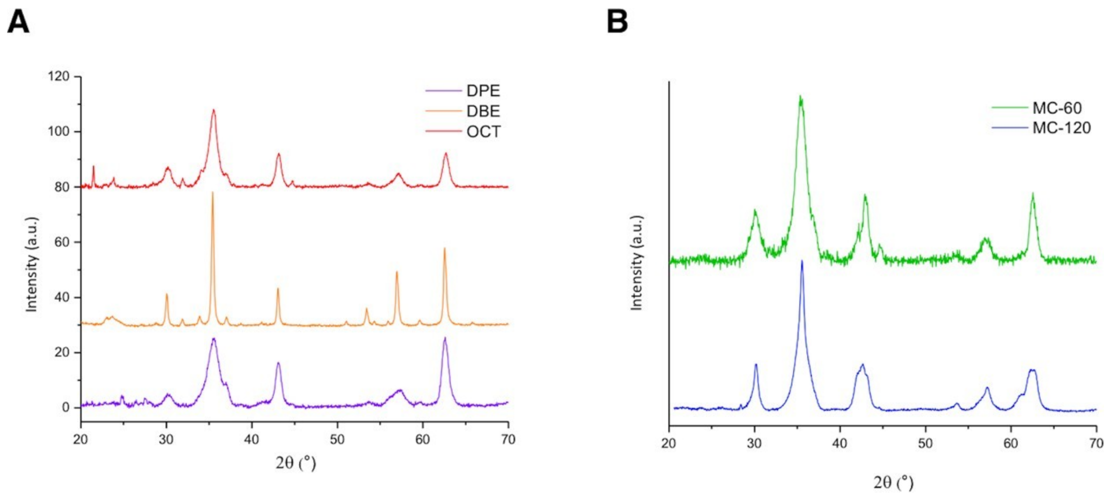

Figure S1. **(A)** Powder X-Ray diffractograms for MNPs synthesized in diphenyl ether (DPE), dibenzyl ether (DBE) and octadecene (OCT). **(B)** Powder X-Ray diffractograms for samples MC-60 and MC-120 synthesised in octadecene after transference to water by ligand exchange.

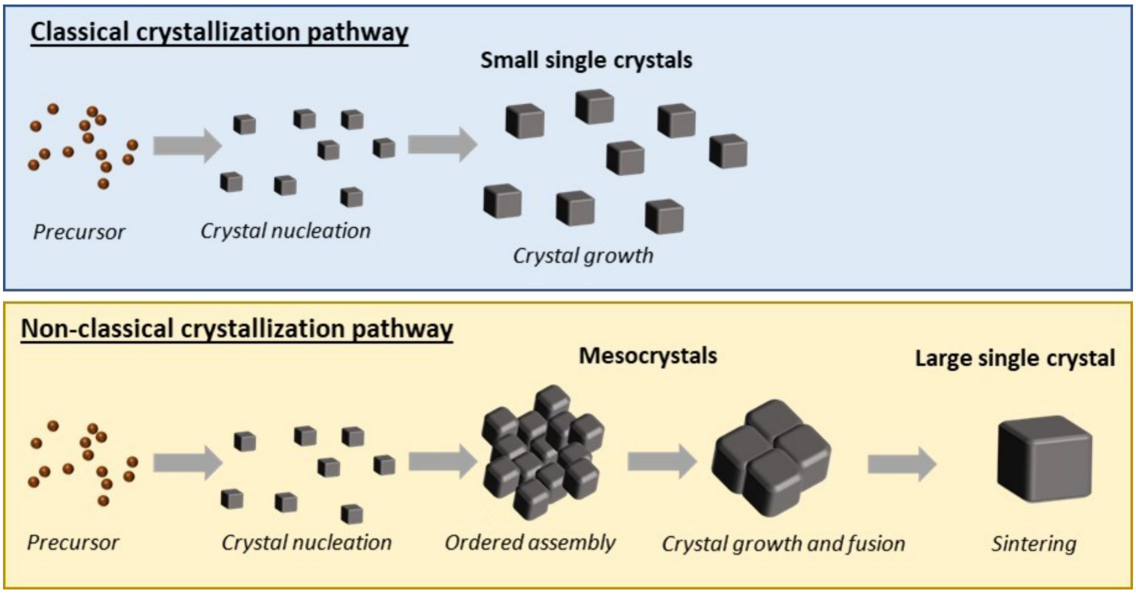

Scheme S1. Classical and non-classical crystallization pathways adapted to the different nanoparticles obtained in this work.

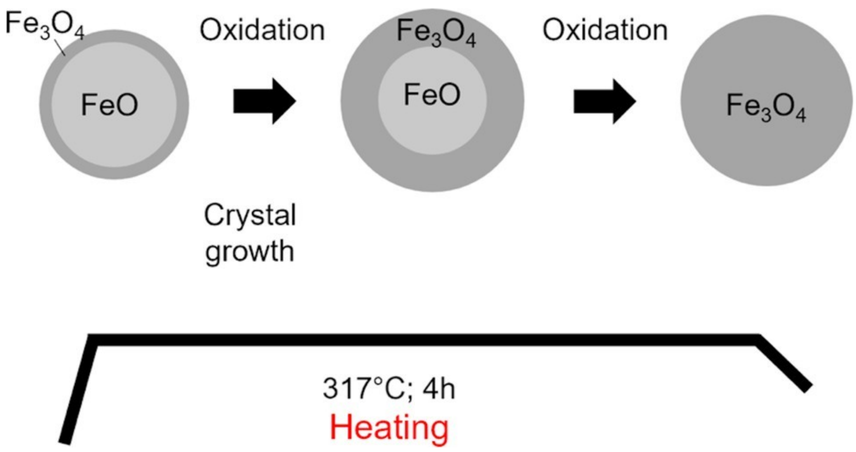

Scheme S2. Representation of the oxidation evolution from wustite to magnetite during the synthesis process for single core nanoparticles as a function of the oleic acid concentration and along the heating, according to previous publications [55].

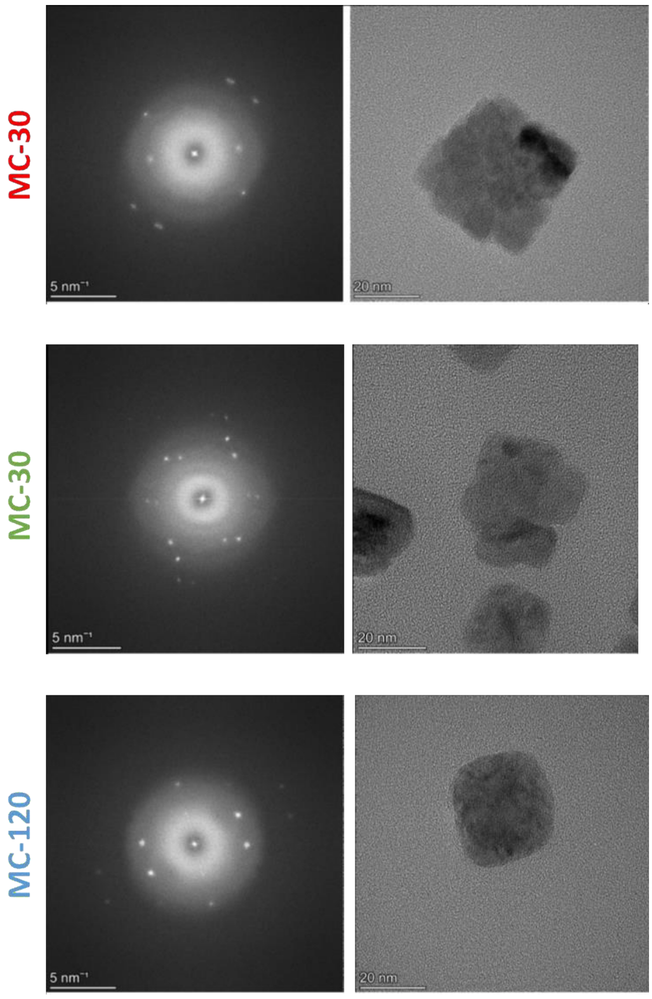

Figure S2. Electron diffraction patterns of samples MC-30, 60 and 120 in organic media.

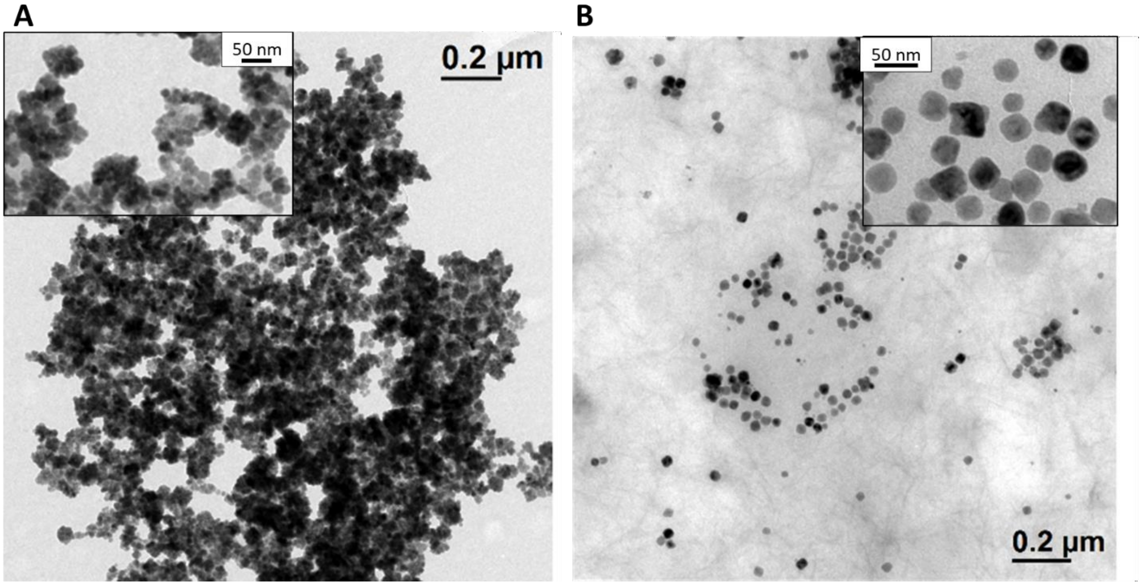

Figure S3. TEM images for DMSA coated **(A)** MC-30 and **(B)** MC-120 in water. Scales bar = 200 nm (main TEM images) and 50 nm (TEM images in squares).

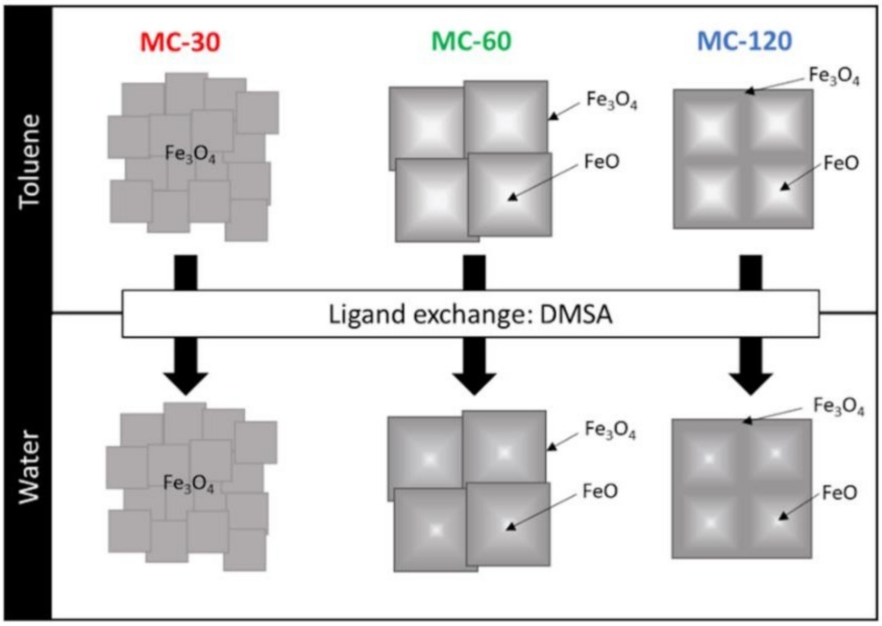

Scheme S3. Upper section: Representation of the wustite inside the magnetite mesocrystals after the synthesis, cooling and washing process, and the conservation in toluene. **Bottom section:** Representation of the oxidation process suffered by the nanoparticles after transference to the aqueous medium through ligand exchange with DMSA.

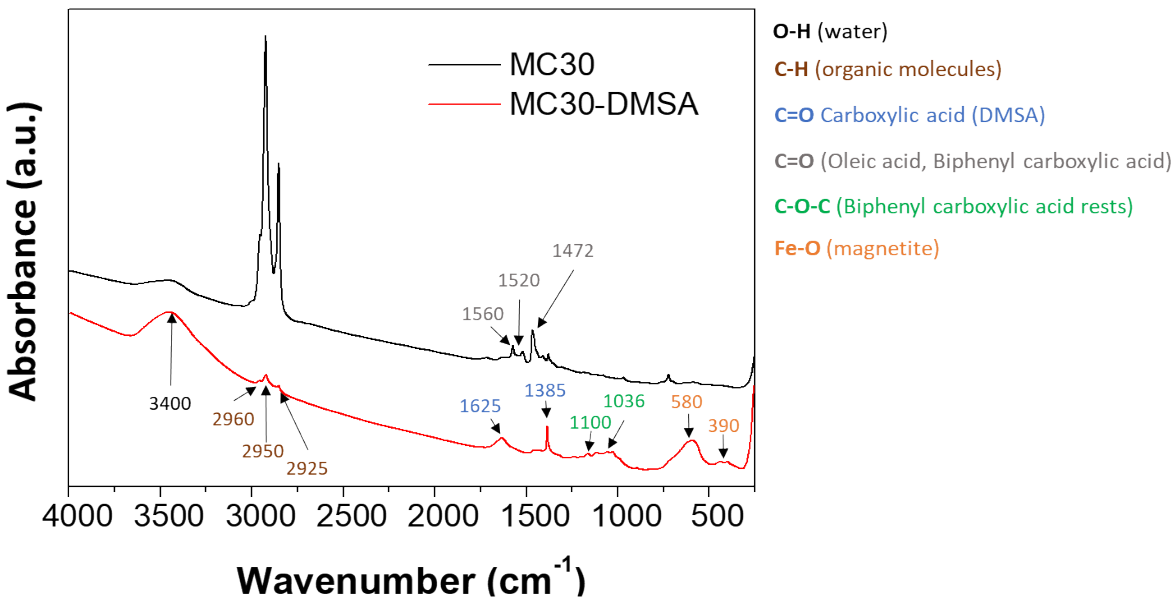

Figure S4. FTIR profiles for MC-30 (black line) and MC-30 – DMSA (red line). The main peaks were assigned as follows: 3400 cm^{-1}: O-H (water); 2960-2925 cm^{-1}: C-H (organic molecules); 1625 and 1385 cm^{-1}: COOH groups of DMSA; 1560 and 1472 cm^{-1}: COOH groups of oleic acid and biphenyl carboxylic acid; 1100 and 1036 cm^{-1}: biphenyl carboxylic acid rests; 580 and 390 cm^{-1}: magnetite (shoulders: maghemite).

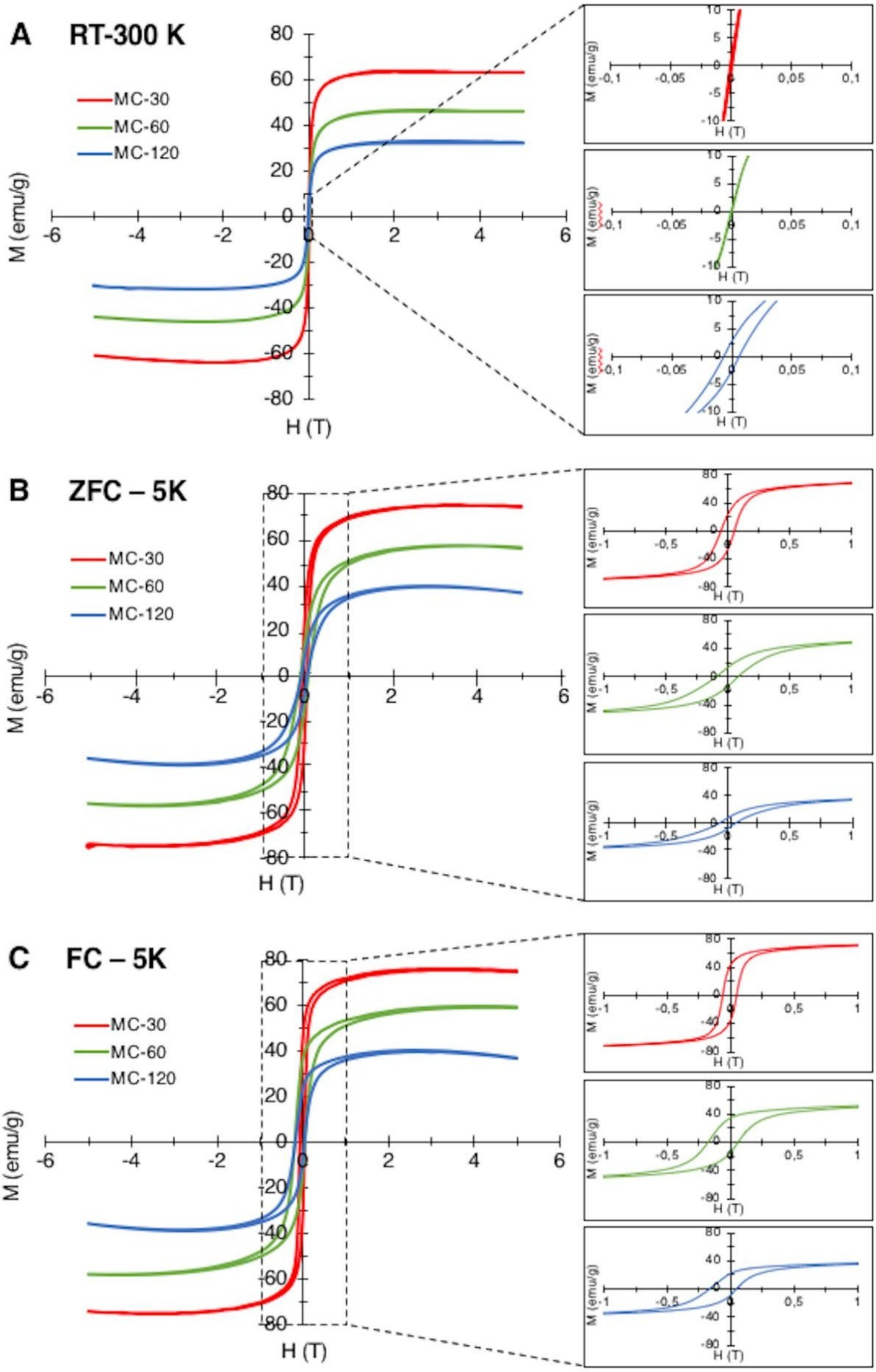

Figure S5. Hysteresis loops for DMSA coated mesocrystals, recorded at **(A)** RT-300 K, **(B)** ZFC-5 K and **(C)** FC-5 K for the MNPs MC-30 (red line), MC-60 (green line) and MC-120 (blue line). Right squares: details of the region (0,0) for each MNP.

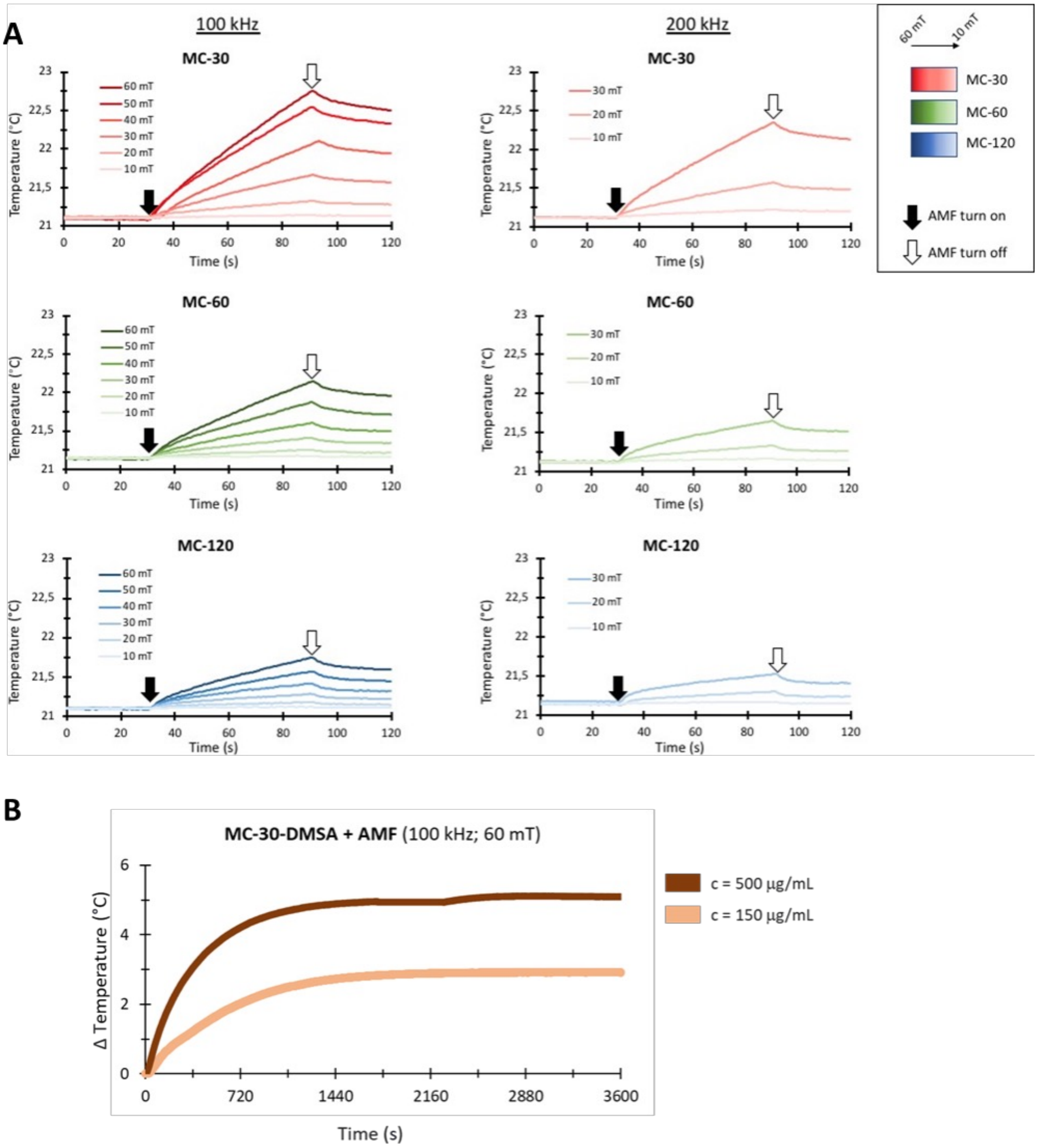

Figure S6. (A) Heating curves for DMSA coated **MC-**30 (red), **MC-60** (green) and **MC-120** (blue) under AMFs of 100 kHz and 10-60 mT (left); and 200 kHz and 10-30 mT (right). Black and white arrows mark the AMF turn on/off. **(B) Heating curves for** DMSA coated **MC-30** at 150 μg Fe/mL (light brown) and c = 500 μg Fe/mL (brown) under AMF of 100 kHz and 60 mT **for 1 hour**.

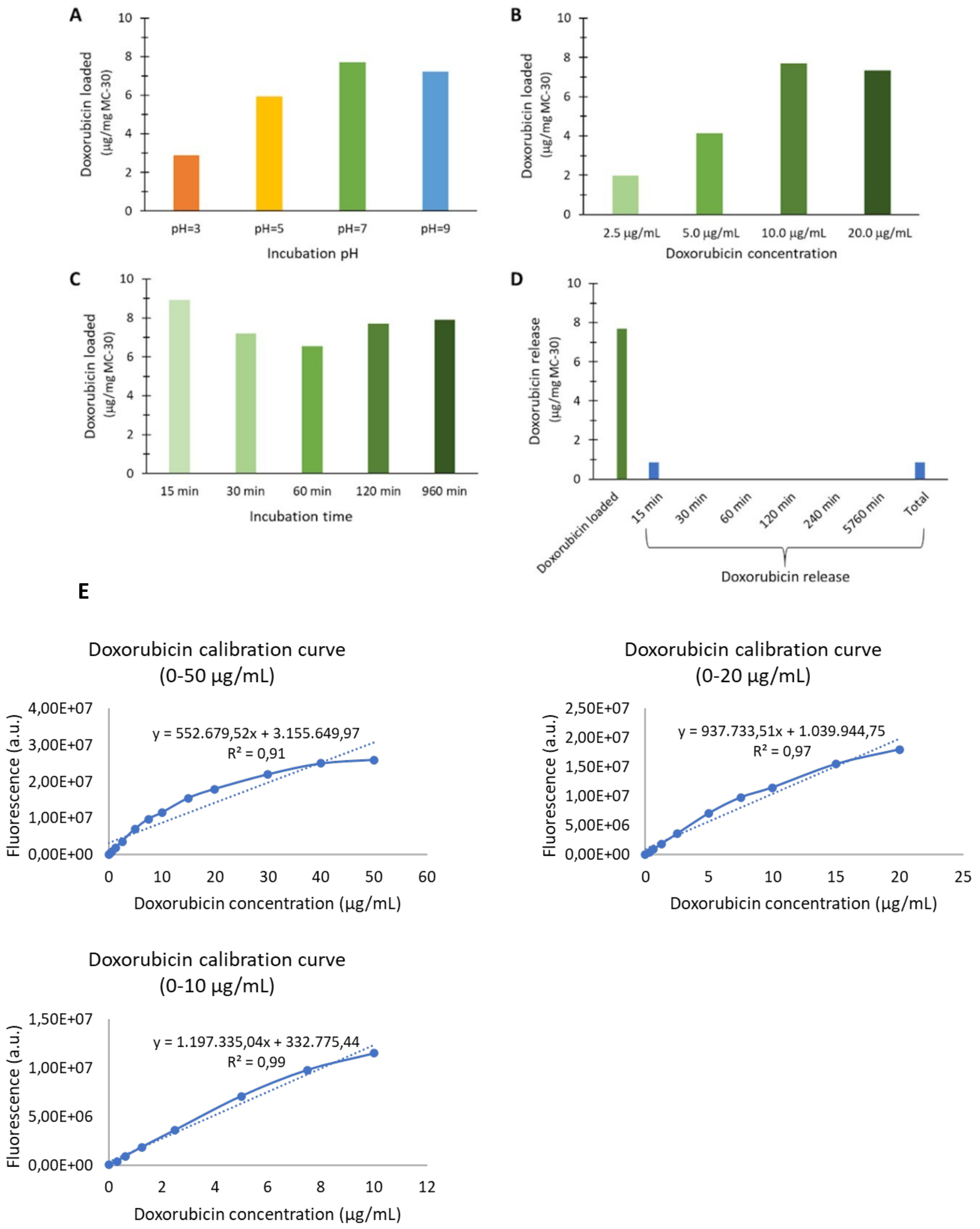

Figure S7. Optimization of doxorubicin loading conditions: (A) pH incubation, **(B)** doxorubicin concentration and **(C)** incubation time. **Doxorubicin release at pH=7 (D)** through successive washings with water at pH=7 at different times (15, 30, 60, 120, 240 and 5760 minutes) after loading at pH=7, 10.0 μg_{doxo}/mL on 1 mg/ml of MNPs for 2 hours. **(E)** Doxorubicin calibration curves.
